# Contribution of reversible histone acetylation to freeze tolerance and recovery in wood frog kidneys

**DOI:** 10.1038/s41598-025-09521-x

**Published:** 2025-07-26

**Authors:** Olawale O. Taiwo, Kenneth B. Storey

**Affiliations:** Institute of Biochemistry, Department of Biology, Carleton University, 1125 Colonel By Drive, Ottawa, ON K1S 5B6 Canada

**Keywords:** Cryopreservation, Epigenetics, Freeze tolerance, Histone modification, *Rana sylvatica*, Epigenetics, Proteomics, Biochemistry, Biotechnology, Cell biology, Molecular biology, Nephrology

## Abstract

The wood frog (*Rana sylvatica*) possesses remarkable adaptation mechanisms that ensures it survival in extreme environmental conditions, including enduring whole body freezing. The present study investigates the epigenetic mechanisms, specifically histone lysine acetylation and deacetylation, which are critical for regulating gene expression and conserving energy, underlying the wood frog’s ability to endure whole-body freezing. We investigated the expression patterns of lysine acetyltransferases (KATs) and lysine deacetylases (HDACs) in wood frog kidney over the freeze-thaw cycle. Our results reveal a significant downregulation of KATs in kidneys of frozen frogs, with specific KATs showing considerable reductions. This suggests that histone acetylation may play a vital role in suppressing gene expression and conserving energy during freezing. Furthermore, histone acetylation marks, including H2AK5ac, H2BK5ac, H3K9ac, H3K23ac, H3K27ac, and H3K56ac, showed repression under frozen and thawed conditions, indicating a role in silencing specific genes. HDACs exhibited dynamic regulation, with HDAC3 and HDAC11 showing significant repression in frozen frog kidneys, while HDAC5, p-HDAC4, and p-HDAC8 were downregulated during the recovery phase, suggesting their involvement in the thawing process. This research provides crucial insights into the epigenetic control of freeze tolerance in wood frog kidneys and offers a foundation for further exploration of epigenetic modifications that mediate the wood frog’s remarkable adaptations for freezing survival.

**Supplementary Information:**

The online version contains supplementary material available at 10.1038/s41598-025-09521-x.

## Introduction

Many organisms struggle to survive extreme cold, but the wood frog (*Rana sylvatica*) is exceptional. These frogs withstand whole-body freezing, allowing ice to form in extracellular spaces and producing high concentrations of glucose as a cryoprotectants to support survival and allow cells and tissues to endure major physiological stresses including ischemia and cell dehydration, and maintaining life despite this severe environmental stress^1,2^. Understanding the epigenetic regulation in renal tissues can consequently shed light on the frog’s overall survival strategy. When frozen, most vital organs of wood frogs cease to function or function sub-optimally; these include kidney, liver, heart and brain^3^. During this process, the wood frog initiates the production of ice-nucleating proteins (INPs) that triggers ice-crystal formation within extra organ spaces and the accumulation and distribution of high concentration of glucose from liver to act as a cryoprotectant^4^. The frogs also undergo metabolic rate depression to conserve energy in the frozen state, significantly reducing energy expensive pathways such as protein synthesis and upregulating protective processes such as production of chaperone proteins and antioxidant defenses to protect tissues from ice damage and activation of stress response pathways^4,5^. The regulation of these processes occurs at multiple biological levels, including transcriptional and post-transcriptional modifications, yet the role of epigenetic mechanisms in this survival strategy remains largely underexplored. Alongside metabolic rate suppression, epigenetic regulation has been implicated in the survival strategies of the wood frog^6–8^.

**Fig. 1:**
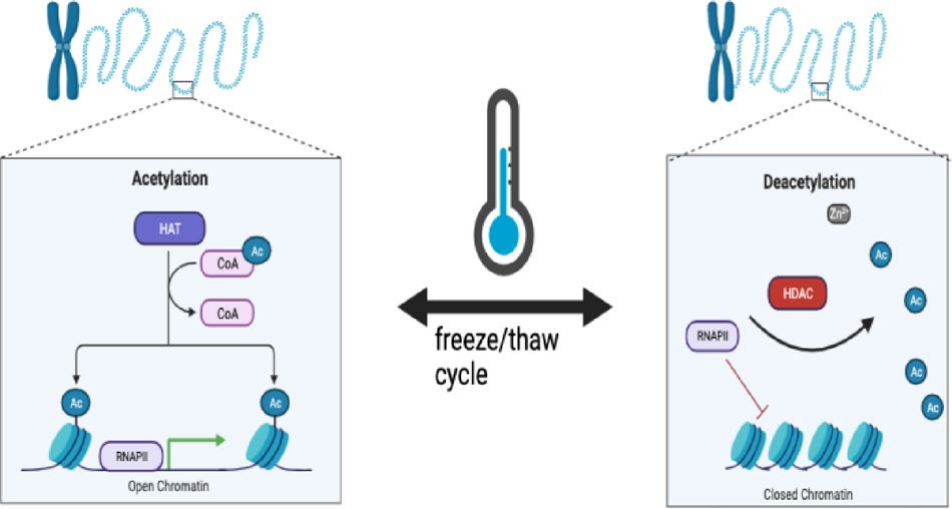
A reversible process of acetylation and deacetylation initiated by the freeze/thaw cycle. HAT (KATs) acetylates lysine in the presence of acetyl co-A to open up the chromatin thereby initiating transcription. On the other hand, HDACs deacetylates histone tails thereby causing a closed chromatin and inhibiting transcription.

Epigenetic modifications regulate biological and physiological processes in response to environmental changes. Recent studies highlight epigenetic regulation as a key factor in managing transcriptional and translational activities under stress conditions^8–10^as seen in wood frogs, that can endure the conversion of about 70% of their body water into extracellular ice throughout winter hibernation^6^. The remarkable ability of some organisms to survive in extreme conditions, such as freezing, relies heavily on the precise regulation of gene expression^11^. One crucial regulatory mechanism is the modification of histones, can impact chromatin structure and gene expression significantly. Lysine acetylation is an important modification where an acetyl group is added to lysine residues on histones mediated by lysine acetyltransferases (KATs) (Fig. 1).

Epigenetic modifications, particularly histone post-translational modifications (PTMs**)**, serve as critical regulators of gene expression in response to environmental stress^6,9^. Among these, histone acetylation is a well-characterized PTM controlled by KATs and histone deacetylases (HDACs), which regulate chromatin accessibility and transcriptional activity^12,13^. Histone acetylation is an ATP-dependent process^14^and its regulation in freeze tolerance may play a key role in energy conservation and transcriptional control. Suppression of acetylation during freezing could reduce unnecessary gene expression and ATP consumption, while its reactivation during thawing may support recovery by enabling the re-expression of genes essential for cellular repair and metabolic reactivation.

It is believed that during freezing, histone modifications allow for the rapid and reversible changes in gene expression necessary for survival^9^. These modifications enable swift repression of non-essential genes and activate protective genes, such as those involved in antifreeze protein production or cryoprotectant synthesis^4^. Epigenetic regulation ensures a flexible yet tightly controlled response to environmental stressors, illustrating the complex interplay between chromatin structure and gene expression believed to be crucial for freeze-tolerant species. These mechanisms can help modify the response to freezing temperatures by silencing genes that are less important or could be detrimental in a frozen state. Furthermore, deacetylation can also play a crucial role in gene regulation by detaching acetyl from tails of histones, a process that can be very important for long term survival in the frozen state. Hence, deacetylation of histones is one mechanism by which wood frogs may achieve tighter gene regulation in the frozen state. By removing acetyl groups from histones residues, specific genes can be effectively silenced^15^preventing excessive energy expenditure on transcription and translation.

Previous studies have highlighted histone methylation dynamics as an important epigenetic mechanism in freeze-tolerant species, including the wood frog^7–9^. However, the role of histone acetylation in this adaptation remains largely unexplored. Studies in mammalian models suggest that HDACs and KATs are involved in metabolic responses to hibernation, ischemia and oxidative stress conditions^10,16^that closely resemble the freeze-induced stress in wood frogs. Furthermore, histone acetylation changes have been implicated in diabetic nephropathy, where hyperglycemia drives modifications in renal tissues^17^. Given that the wood frog also experiences glucose accumulation and kidney stress during freezing^18,19^it is likely that histone acetylation dynamics in the kidney mirror regulatory pathways observed in diabetic nephropathy and other metabolic disorders.

In this study, we investigate the expression patterns of KATs, HDACs, and histone acetylation marks in wood frog kidneys across control, frozen, and thawed conditions. We hypothesize that acetylation will be repressed in the frozen state, consistent with metabolic suppression, and that thawing will lead to its restoration, facilitating transcriptional reactivation. By examining these histone modifications at the protein level, this study aims to expand our understanding of epigenetic regulation in freeze tolerance and provide broader insights into how histone modifications contribute to metabolic adaptation in vertebrates. By investigating the expression patterns of seven lysine acetyltransferases and ten lysine deacetylases in kidneys from frozen *R. sylvatica*, the current study provides insights into the potential regulatory mechanisms involved in kidney function in response to freezing and recovery from freezing. To examine the responses of KATs and HDACs to freezing, relative levels of these enzymes were assessed in wood frog kidneys from control, frozen and thawed conditions along with changes in the relative levels of key histone acetyl-lysine modifications.

## Results

### Protein amounts of KATs under control, frozen and thawed conditions in wood frog kidneys

**Fig. 2:**
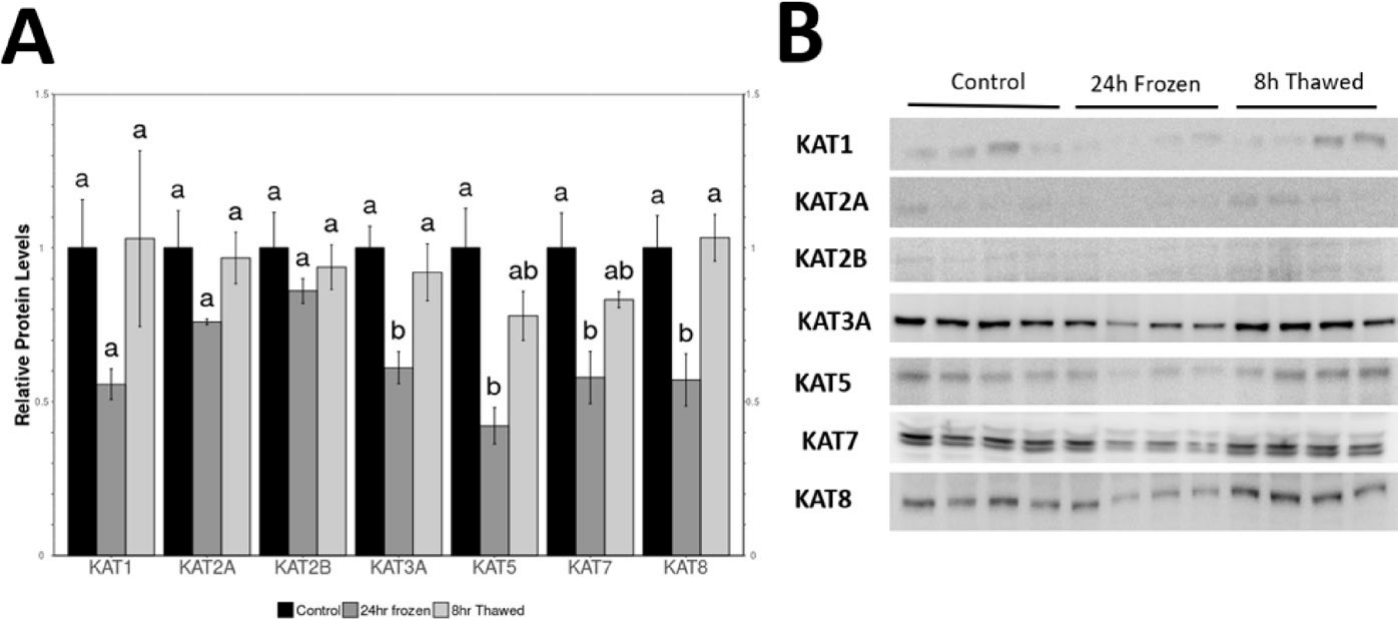
Relative protein levels of 7 lysine acetyl transferases (KATs) in *R. sylvatica* kidneys as determined by Western immunoblotting. (**A**) Histogram showing mean (± SEM, n=4) standardized expression levels of KAT1, KAT2A, KAT2B, KAT3A, KAT5, KAT7, KAT8 under control, 24 h freezing and 8 h thawed conditions. Data are mean ± SEM (*n* = 4 independent trials). Statistical significance for freezing and thawing values, relative to the standardized control, was determined using one-way analysis of variance (ANOVA) with Tukey’s post hoc test where a, b, c that share the same letters were not statistically different from one another (*—*p* < 0.05). (**B**) Representative western blots for individual KMTs under each experimental condition. Original full blots are presented in Supplementary Figure S1.

Levels of KAT1, KAT2A, KAT2B, KAT3A, KAT5, KAT7 and KAT8 were evaluated via immunoblotting of extracts from wood frog kidney under 3 conditions which are the control, 24 h frozen, and 8 h thawed groups. Expression of all KATs followed a similar pattern of reduced levels in tissues from 24 h frozen frogs, as compared with controls. However, only KAT3A, KAT5, KAT7 and KAT8 levels from frozen frogs reached statistical significance, falling below 60% of the control values. The level decreased in 24 h Frozen recovered to the control level in 8 h Thaw suggesting that the effects of freezing were reversed quite quickly after thawing (Fig. 2).

**Fig. 3:**
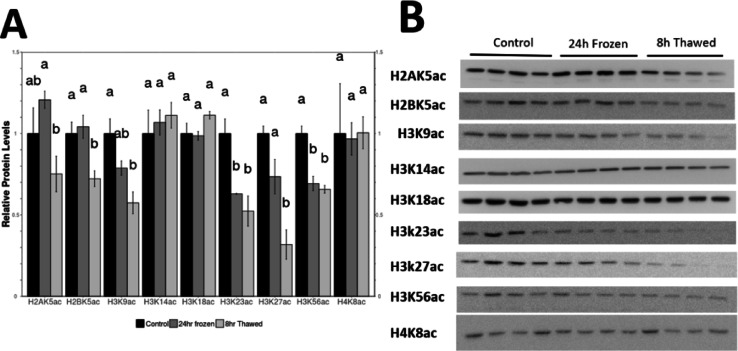
Relative protein levels of 9 acetylated histone residues in *R. sylvatica* kidneys as determined by Western immunoblotting. (**A**) Histogram shows mean (± SEM, n=4) standardized expression levels of H2AK5ac, H2BK5ac, H3K9ac, H3K14ac, H3K18ac, H3K23ac, H3K27ac, H3K56ac and H4K8ac under control, 24 h freezing and 8 h thawed conditions. (**B**) Representative western blots for individual histone marks under each experimental condition. Original blots are presented in Supplementary Figure S1. Data are mean ± SEM (*n* = 4 independent trials). Statistical significance for freezing and thawing values, relative to the standardized control, was determined using one-way analysis of variance (ANOVA) with Tukey’s post hoc test where a, b, c that share the same letters were not statistically different from one another (*—*p* < 0.05).

### Analysis of acetylated histone levels in control, frozen and thawed States

Lysine acetylation on H2A, H3B, H3 and H4 proteins was measured by evaluating the relative modifications levels of 9 acetylated histone marks. H2AK5ac, H2BK5ac, H3K9ac, H3K14ac, H3K18ac, H3K23ac, H3K27ac, H3K56ac, H4K8ac in histone protein extracts of *R. sylvatica* kidney from control, 24 h frozen and 8 h thawed groups (Fig. 3). Histone marks showed a range of changes from slight repression in frozen and thawed kidney samples to strong significant repression in frozen and thawed samples, as compared to the controls. The most notable changes were seen in H2BK5ac, H3K9ac, H3K23ac, H3K27ac, and H3K56ac showing significant repression primarily in the tissues after recovery from freezing (Fig. 3).

**Fig. 4:**
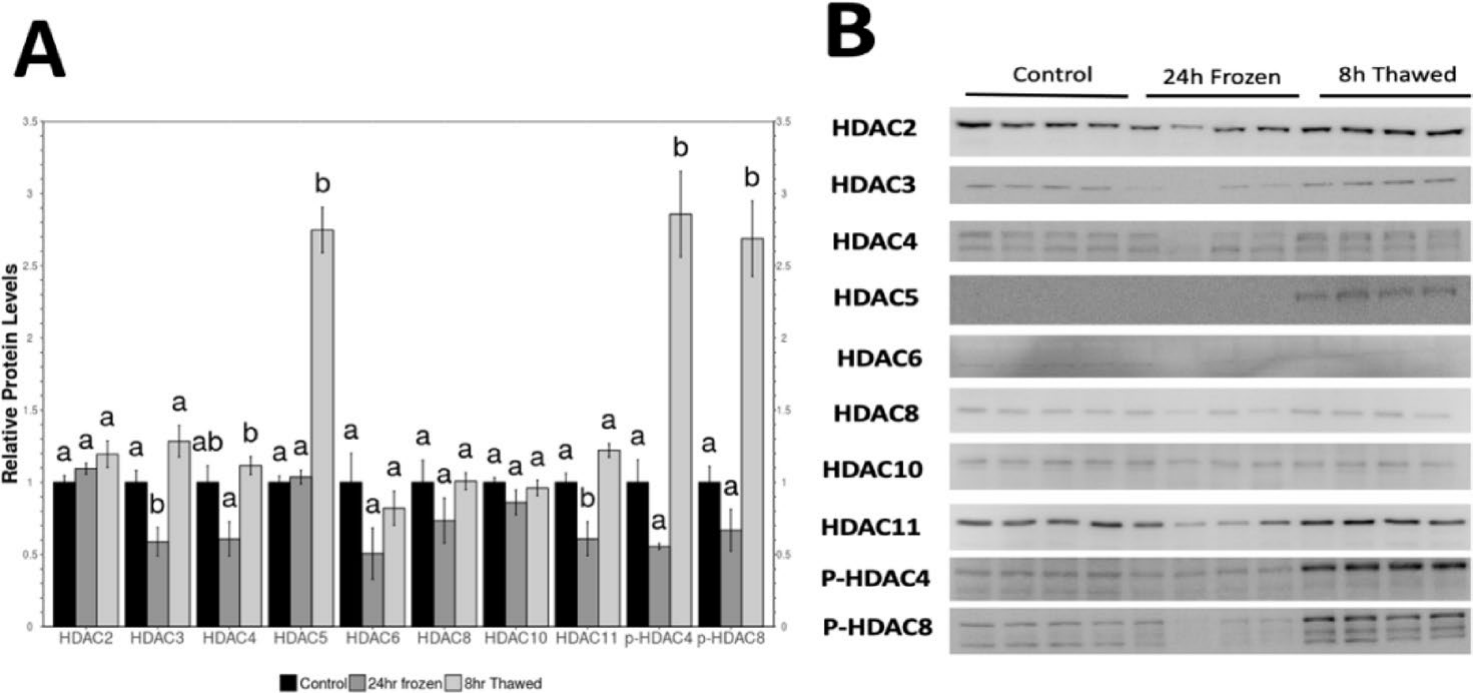
Relative protein levels of histone lysine deacetylases in *R. sylvatica* kidneys as determined by Western immunoblotting. (**A**) Histogram showing mean (± SEM, n=4) standardized expression levels of control, 24 h freezing and 8 h thawed conditions. For each HDAC, values that share the same letter designation are not significantly different from one another, while values with different letter designation are significantly different (p < 0.05). (**B**) Representative western blots for individual HDACs as well as p-HDAC4 and p-HDAC8 under each experimental condition. Data are mean ± SEM (*n* = 4 independent trials). Statistical significance for freezing and thawing values, relative to the standardized control, was determined using one-way ANOVA with Tukey’s post hoc test where a, b, c that share the same letters were not statistically different from one another (*—*p* < 0.05). Original full blots are presented in Supplementary Figure S1. .

### Expression of HDACs in frozen, thawed and recovery phases

Levels of lysine deacetylase enzymes (HDAC2, HDAC3, HDAC4, HDAC5, HDAC6, HDAC8, HDAC10, HDAC11, p-HDAC4, p-HDAC8) levels were evaluated via immunoblotting of extracts of wood frog kidney tissue from control, 24 h frozen, and 8 h thawed conditions (Fig. 4). Changes in the expression of HDAC3, HDAC5, HDAC11, p-HDAC4 and p-HDAC8 all reached statistical significance changes. HDAC3 and HDAC11 were showed statistical repression in the kidney of 24 h frozen frogs, compared to controls, but levels rebounded again after thawing to values not significantly different from controls. By contrast, HDAC5, p-HDAC4 and p-HDAC8 showed no significant changes between control and 24 h frozen states but levels increased strongly after 8 h thawed reaching values over 2.5 fold higher than controls (Fig. 4).

## Discussion

Wood frogs (*Rana sylvatica*) possess remarkable adaptations that enable them to survive extreme environmental conditions, including freezing temperatures, that pose significant challenges to most other organisms. Their ability to endure whole body freezing necessitates the suppression of non-essential cellular functions, including transcription and translation, while maintaining mechanisms critical for survival, such as metabolic rate depression and cryoprotectant accumulation^2^. This current research focuses on histone acetylation and deacetylation in the wood frog kidney where protein levels were measured to gain more knowledge into the epigenetic mechanisms contributing to freeze tolerance.

Our findings reveal distinct histone modification patterns in wood frog renal tissue, particularly in the regulation of KATs, HDACs, and their corresponding histone marks. Specifically, we observed significant downregulation of KAT3A, KAT5, KAT7, and KAT8 in frozen kidneys (Fig. 2), coinciding with a significant reduction in H3K23ac and H3K56ac levels (Fig. 3). These changes suggests that suppression of HAT activity contributes to global repression of transcriptional activity as part of an energy-conservation strategy, consistent with previous observations that transcriptional repression is a key component of freeze tolerance^9,10,16^. Since histone acetylation is an ATP-dependent process, its downregulation may serve to minimize energy expenditure while ensuring genome stability under extreme physiological stress. Interestingly, the suppression of histone acetylation persisted into the thawed state, suggesting that transcriptional reactivation may be delayed. The reduced levels of H2BK5ac, H3K9ac, H3K23ac, H3K27ac, and H3K56ac in thawed kidneys imply that gene expression remains suppressed during early recovery, possibly to allow for chromatin stabilization before full transcriptional reactivation. This aligns with findings in hibernating mammals, where post-arousal recovery requires controlled gene reactivation to prevent oxidative stress and DNA damage^16^. Similarly, the reduced levels of H3K56ac, a mark associated with DNA repair and replication^20^align with the metabolic suppression observed in freeze tolerance, where cell cycle activity is minimized to prevent errors during stress conditions. Further studies examining longer post-thaw time points could provide valuable insight into the timeline of epigenetic homeostasis restoration.

The regulation of HDACs also followed distinct patterns across the freeze/thaw cycle. HDAC3 and HDAC11 were significantly downregulated in frozen kidneys, mirroring the suppression of transcription-associated histone acetylation marks. HDAC3, a key regulator of transcriptional repression and genome stability^21^may be downregulated to enable selective expression of stress-response genes, despite the overall transcriptional suppression. Similarly, HDAC11, known for its role in metabolic regulation^22^may be suppressed to prevent excessive deacetylation, thereby maintaining chromatin stability during freezing stress. HDAC3 is known to regulate H3K9ac, H3K14ac, H4K5ac and H4K12ac^23,24^and its inhibition typically results in increased acetylation of these marks (Bhasira et al. 2010). However, our result do not show the expected increase in these marks following HDAC3 downregulation, suggesting that alternative compensatory mechanisms may be involved. One possibility for H3K9ac is that SUV39H1-mediated methylation at H3K9 competes with acetylation at the same site, thereby precluding H3K9ac even in the presence of HDAC3 suppression^25,26^. Also, HDACs, like HDAC5 and HDAC11, contribute to histone deacetylation, balancing out the effect of HDAC3 suppression^27^. This cross-talk between histone modifications has been demonstrated in various mammalian systems^28^ and may apply to stress-adapted species as well. Additionally, KAT2A activity is known to be regulated via phosphorylation, and stress-induced post-translational modifications may impair its function even if the protein is present^29,30^. Furthermore, freezing imposes metabolic constraints, including reduced availability of acetyl-CoA due to glucose diversion toward cryoprotectant synthesis^2,31,32^. This substrate limitation may restrict the acetylation capacity of KATs, helping to explain the lack of expected acetylation increases. It is also important to consider biological variability across individual frogs as they may exhibit slight differences in acetylation patterns due to metabolic status or differential expression of epigenetic regulators.

Conversely, HDAC5, phospho-HDAC4, and phospho-HDAC8 were significantly upregulated in thawed kidneys. These class IIa HDACs are known to shuttle between the nucleus and cytoplasm in response to stress and metabolic signaling^33^. The upregulation of phospho-HDAC4 and phospho-HDAC8 indicates their cytoplasmic sequestration, which may allow for a selective reactivation of transcriptional programs during recovery while preventing premature gene activation that could compromise genome stability. Also, the phosphorylation of HDAC4 and HDAC8 suggests their activation in a non-histone regulatory role, possibly in cytoplasmic signaling pathways linked to tissue repair and metabolic recovery^34^. Previous studies have shown that HDAC5 plays an important role in skeletal muscle regeneration and metabolic adaptation^35^suggesting that its upregulation post-thaw may contribute to recovery processes such as mitochondrial biogenesis and gluconeogenesis^36–38^. These findings indicate that HDAC activity extends beyond histone modifications, influencing broader metabolic and cellular pathways that support recovery from freezing stress. One important factor that is to be considered is that HDACs are not restricted to deacetylation and histone modifications as they are known to modify non-histone proteins^39^. This means that some deacetylases that were significantly altered may be modifying other non-histone proteins that are involved in other processes like metabolic regulation or DNA repair^35,39,40^.

Another important consideration is the biological relevance of histone acetylation dynamics beyond the context of freeze tolerance. While previous studies have drawn parallels between histone modifications in freeze-tolerant organisms and cancer models^10,41,42^our findings suggest that a comparison may be made with metabolic disorders such as diabetic nephropathy. Hyperglycemia, a hallmark of diabetic kidney disease, has been shown to drive histone acetylation changes in renal tissues^17^. Given that wood frogs experience glucose accumulation as a cryoprotectant^19,43,44^it is believable that the regulation of HDACs and KATs in the kidney may parallel epigenetic changes seen in diabetes. This alternative framework provides a new avenue for exploring the translational relevance of freeze tolerance mechanisms in human metabolic diseases.

Taken together, our findings highlight the critical role of epigenetic modifications in balancing genome stability and metabolic demand during freeze tolerance. The coordinated regulation of HDACs, KATs, and their epigenetic marks suggest a controlled mechanism for transcriptional suppression during freezing and regulated reactivation upon thawing. This study provides the first detailed characterization of histone acetylation changes in wood frog kidneys, expanding our understanding of how epigenetic regulation facilitates adaptation to extreme environmental stress. Future investigations should explore the downstream gene targets of these modifications, their interactions with other epigenetic regulators such as DNA methylation, and the broader implications of these findings for vertebrate stress physiology and cryopreservation strategies. Additionally, investigating the non-histone targets of these HDACs during freeze-thaw cycles, as well as the specific signaling pathways regulated by their phosphorylation states.

Together, these findings emphasize the crucial role of epigenetic modifications in balancing genome stability and metabolic demand during freeze tolerance. By modulating histone acetylation and deacetylation, the wood frog effectively silences energy-intensive processes, preserving cellular integrity under extreme environmental conditions. However, our current study marks the foundational investigation into the role of lysine acetyltransferases and lysine deacetylases in the freeze-tolerant wood frog. While this study focuses on protein-level regulation, it is important to acknowledge the potential role of transcriptional changes in HDAC and KAT expression. RNA-seq or qPCR validation could provide additional insights, but histone modifications are often regulated independently of mRNA levels, as acetylation changes can occur rapidly in response to environmental stimuli. Given the immediate and reversible nature of histone acetylation, examining protein-level changes offers a direct perspective on its functional role during freeze tolerance. Future studies should incorporate RNA-seq and ChIP analyses to precisely identify gene targets and chromatin states regulated by these histone modifications.

## Materials and methods

All animals used in this experiment were cared for in accordance with the guidelines of the Canadian Council on Animal Care and experimental procedures had the prior approval of the Carleton University Animal Care Committee (protocol #106935; 11 June 2017). All methods are reported in accordance with ARRIVE guidelines.

### Animal collection

Male wood frogs (*R. sylvatica*) weighing roughly 5–7 g were collected in early spring from melt-water ponds around Ottawa, Ontario, Canada. Following which, a transfer of the animals to Carleton University was done using coolers containing fragmented ice/snow. In the lab, the frogs were bathed briefly using tetracycline and placed in boxes containing damp sphagnum moss. All Frogs were acclimated for at least 1 week in an incubator at 5 °C (a temperature close to that of the forest ponds). Subsequently, the control group of frogs were sampled from this total number. For freezing exposure, remaining frogs were placed in closed plastic containers that were each padded with a sheet of moist paper towel and were then moved into an incubator at −4.0 °C for 45 min. This exposure time dropped frog body temperatures to below zero and triggered ice nucleation across the skin. Temperature was then raised to −2.5 °C and frogs were held in this state for 24 h. Subsequently, 50% of frogs that were frozen frogs were selected for sampling randomly. The remainder of the frogs were taken back to the 5 °C incubator for 8 h and allowed to thaw and recover and were then sampled from this condition. For testing, control, 24 h frozen, and 8 h thawed frogs were sacrificed by pithing at which point kidneys were quickly excised and immediately frozen using liquid nitrogen. Tissues were kept at a temperature of −80 °C until use. All animals used in this experiment were cared for in accordance with the guidelines of the Canadian Council on Animal Care and experimental procedures had the prior approval of the Carleton University Animal Care Committee (protocol #106935; 11 June 2017).

### Total protein isolation

Total protein was extracted from samples of frozen kidney (~ 50 mg each) from 4 individuals for each condition (control, frozen, thawed) as previously described^9,45^. After weighing, tissues were powdered using mortar and pestle while ensuring cooling in liquid nitrogen. With the use of P10 homogenizer, these samples were then quickly homogenized 1:5 w: v in pre-cooled buffer containing 20 mM HEPES, 200 mM NaCl, 0.1 mM EDTA, 10 mM NaF, 1 mM Na_3_VO_4_, and 10 mM β-glycerophosphate at pH 7.4 and a few drops of protease inhibitor phenylmethylsulfonyl fluoride (PMSF) with 1 µL/mL of a protease inhibitor cocktail containing AEBSF, aprotinin, bestatin, E64 & leupeptin (Catalogue # 808 PIC001.1; BioShop Canada Inc., Burlington, ON, Canada).

The homogenized samples were then centrifuged at 10,000×g for 15 min at 4 °C, and the supernatant with soluble proteins was collected. Protein concentration was measured using the BioRad protein assay (Catalogue #500-0002; BioRad Laboratories, Hercules, CA, USA) at 595 nm on an MR5000 microplate reader (Dynatech Laboratories, Chantilly, VA, USA). Following that, all samples were adjusted to 10 µg/µL protein concentration, then mixed 1:1 v: v with Tris buffer containing sodium dodecyl sulfate (SDS): 100 mM Tris-base, 4% w/v SDS, 20% v/v glycerol, 0.2% w/v bromophenol blue, and 10% v/v 2-mercaptoethanol, pH 6.8 for a final concentration of 5 µg/µL protein per sample. All samples were denatured by heating in boiled water bath for 10 min and stored at − 40 °C until ready for use.

### Histone isolation

Histone proteins were isolated from frozen samples of kidney from the control, 24 h frozen, and 8 h thawed frogs with *n* = 4 following a detailed procedure previously described by^7^. Briefly, samples were homogenized in Triton Extraction Buffer (TEB), centrifuged, and treated with 0.2 M H_2_SO_4_. Histones were precipitated with trichloroacetic acid (TCA), washed with acetone, and air-dried. The histone pellet was dissolved in water, sonicated, and quantified. Validation was done via immunoblotting to ensure proper isolation and quality.

### Western Immunoblotting

Equal amounts of protein samples from the kidneys of control, 24-hour frozen, and 8-hour thawed frogs (∼30 µg/mL based on the target protein) were loaded into 6–15% SDS-polyacrylamide gels. The percentage of acrylamide in the resolving gel was determined by the molecular weight of the target protein. Additionally, 3 µL of BLUeye prestained protein ladder (10–245 kDa) was loaded in other lanes for molecular weight reference. The upper stacking gel (pH 6.8) consisted of 5% acrylamide in 1 M Tris buffer with 0.1% SDS, 0.1% APS, and 0.1% TEMED, while the resolving gel (pH 8.8) ranged from 8 to 15% acrylamide in 1.5 M Tris buffer with the same SDS, APS, and TEMED concentrations. The gels were run for 30–180 min at 180 V using a BioRad Mini Protean III system in a running buffer of Tris-base, glycine, and SDS.

After electrophoresis, proteins were transferred onto 0.45 μm PVDF membranes via electroblotting at room temperature for 45–180 min at 160 mA in transfer buffer (Tris-base, glycine, methanol). To block non-specific antibody binding, membranes were incubated in TBST buffer containing either skimmed milk (1–10%) for 30 min or polyvinyl alcohol for 30–90 s. Primary antibodies (1:1000 dilution in TBST) were applied overnight at 4 °C to detect relative protein levels of lysine acetyltransferases (KATs), histone deacetylases (HDACs) and their acetyl marks. Following this, membranes were washed and incubated with HRP-conjugated secondary antibodies ((1:8000 v: v in TBST; Cat#APA002P, BioShop Canada Inc.) at room temperature for 30 min. After washing, the membranes were visualized using chemiluminescence and a Chemi-Genius Bio Imaging System (original uncropped blot images are presented in supplementary files S1). Finally, membranes were stained with Coomassie blue to standardize protein loading.

Antibodies probed in this study were procured from different commercial suppliers and their names and catalog numbers are listed below. ABclonal Technology supplied antibodies against KAT3A (A1334), KAT5 (A1678); KAT7 (A5823); HDAC8 (A5829); HDAC9 (A1516), p-HDAC4 (AP0280), p-HDAC8 (AP0360), H3K56ac2 (A7256), H2BK5ac (A15621), H3K14ac (A7254), H2AK5ac (A15620), H3K27ac (A7253) and H3K18ac (A7257 antibodies. Furthermore, Abbexa supplied HDAC5 (abx326108) and HDAC10 (abx317878) antibodies. Cell Signaling Technology supplied antibodies against HDAC1 (#345897); HDAC2 (#571567); HDAC3 (#850577); HDAC7 (#2882); H3K18ac (#9675); H2AK5ac (#2576P) and H3K23ac (#96745). Genescript supplied HDAC4 (A00429) and GeneTex supplied antibodies against KAT1 (#GTX110643); KAT2A (#GTX114428); KAT2B (GTX109666); KAT8 (GTX129380) and HDAC6(GTX5380).

### Statistical analysis

Chemiluminescent protein bands from imaged blots were measured through band intensity quantification using the ChemiGenius Bio Imaging System with GeneTools Software (Syngene, Frederick, MD, USA). The densities of these bands were normalized against the intensity of Coomassie blue-stained bands from the same lane, ensuring that the selected bands did not display differential expression among conditions and were distinct from the immunoblot band of interest. Data for each experimental condition are presented as mean ± SEM, with *n* = 4 samples taken from different animals. Statistical analysis was conducted using a one-way ANOVA followed by Tukey’s post-hoc test, with significance defined as *p* < 0.05, using the RBioPlot statistical package^46^.

## Conclusions

In summary, our study revealed tissue-specific alterations in lysine acetyltransferases (KATs), histone deacetylases (HDACs), and the histone lysine marks that they acetylate in response to the freeze/thaw cycle of freeze tolerant wood frogs. At the core of the wood frog’s freezing survival strategy lies the reallocation of limited fuel/energy reserves accumulated before temperatures plummet. This allocation is enabled through intricate controls, primarily involving transcriptional regulation, that usher the frog into a state of hypometabolism. Differentially regulated lysine acetylation patterns may form the foundation for a downregulation of non-essential pathways during freezing while concurrently preserving pathways that are crucial for survival. However, it is essential to acknowledge the limitations of our results. Global changes in histone modifications signify overarching trends across the genome, yet the precise translation of these global alterations to individual genes remains a principal avenue for future research. Establishing direct connections between changes in histone modifications at the gene level is vital for expanding our understanding of the complex roles played by histone modifications and how they support kidney (and potentially other organs) during wood frog’s remarkable capacity for whole-body freezing survival.

## Electronic supplementary material

Below is the link to the electronic supplementary material.

Supplementary Material 1

Supplementary Material 2

Supplementary Material 3

## Data Availability

All data that support the findings of this study are available from the corresponding author upon request.
