# Supplementary material for: Contribution of reversible histone acetylation to freeze tolerance and recovery in wood frog kidneys
Source: Sci Rep. 2025 Jul 26;15:27243. doi: 10.1038/s41598-025-09521-x (PMC12297572; doi:10.1038/s41598-025-09521-x)

Supplemental Files

Figure 2 full blot images

FIG. 2 KAT1

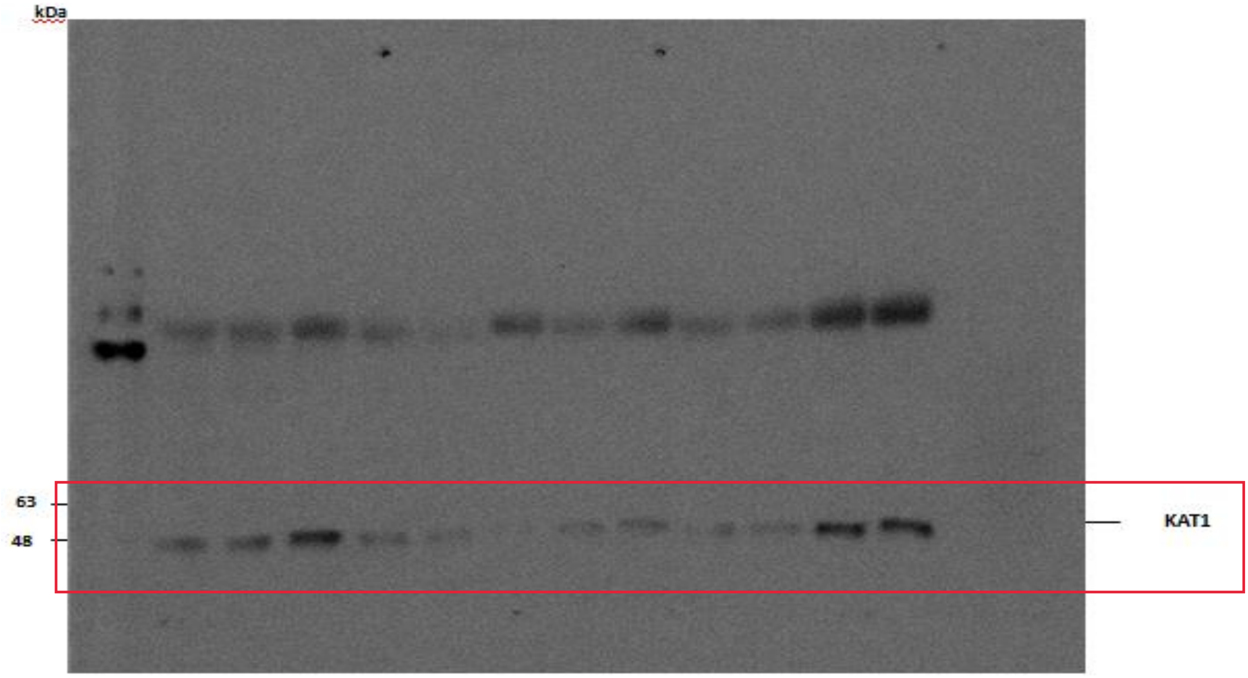

FIG. 2 KAT2A

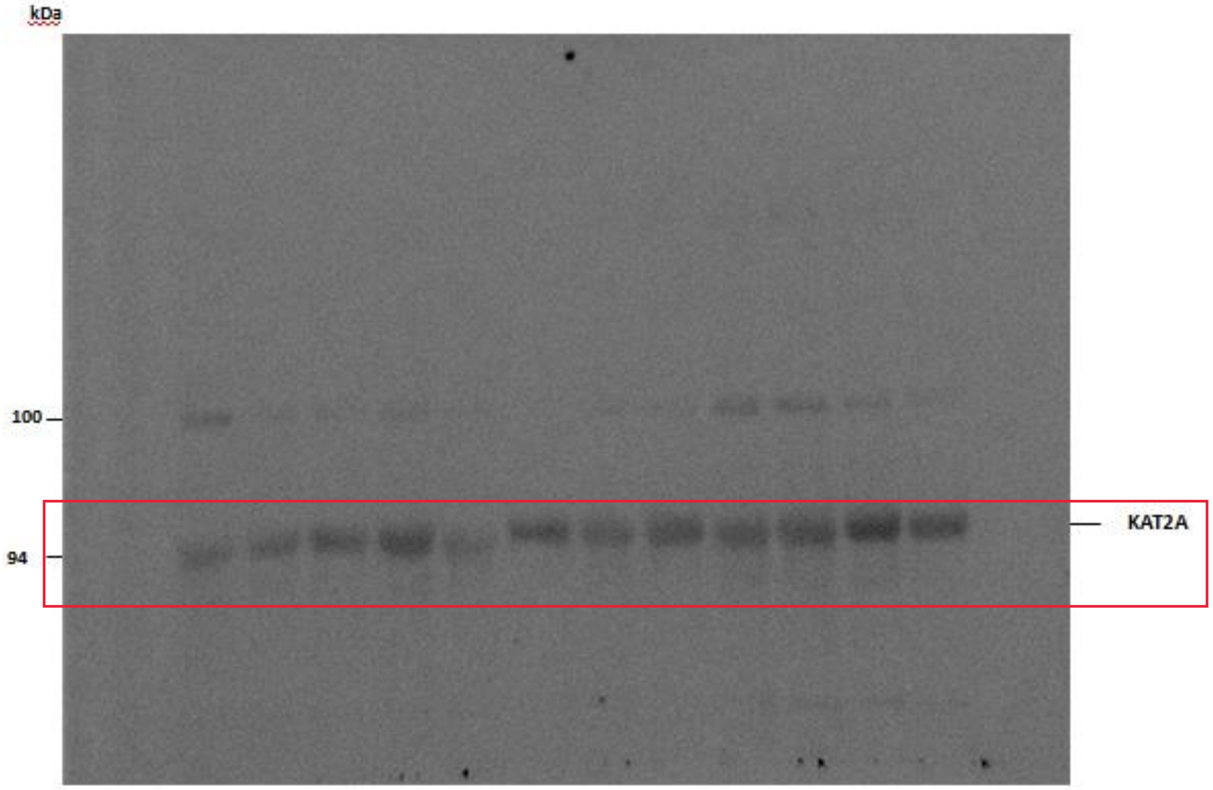

FIG. 2 KAT2B

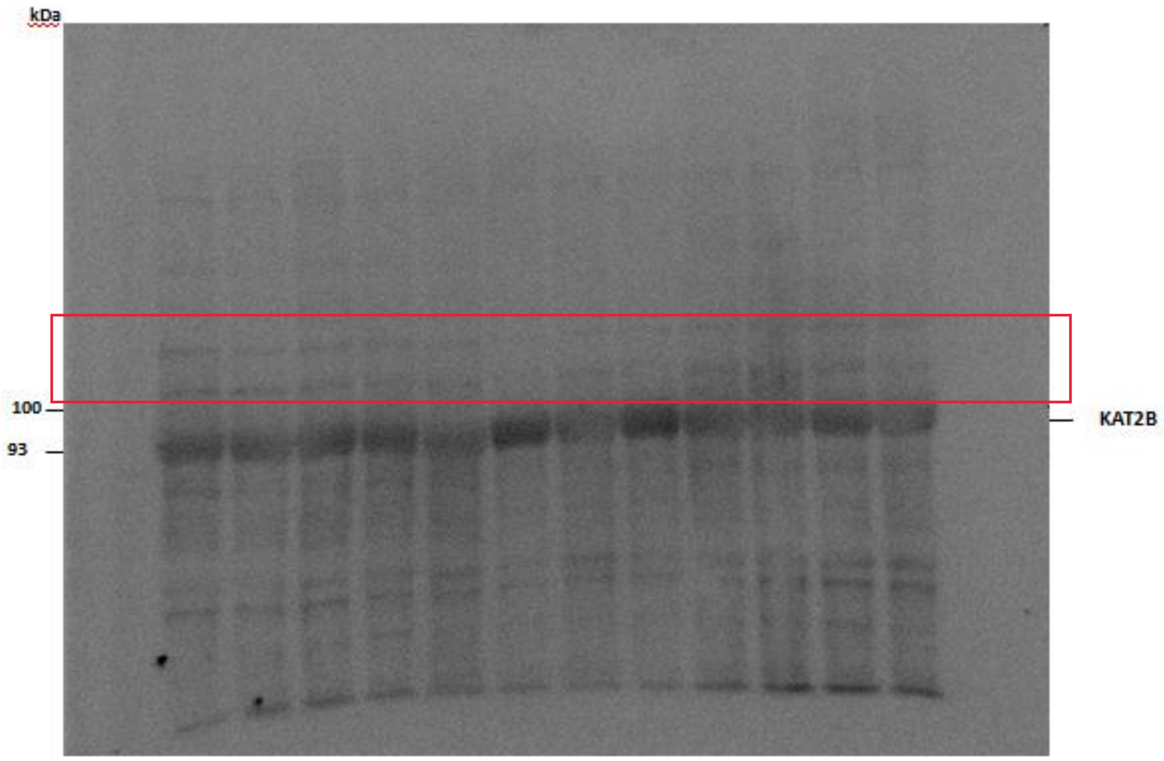

FIG. 2 KAT3A

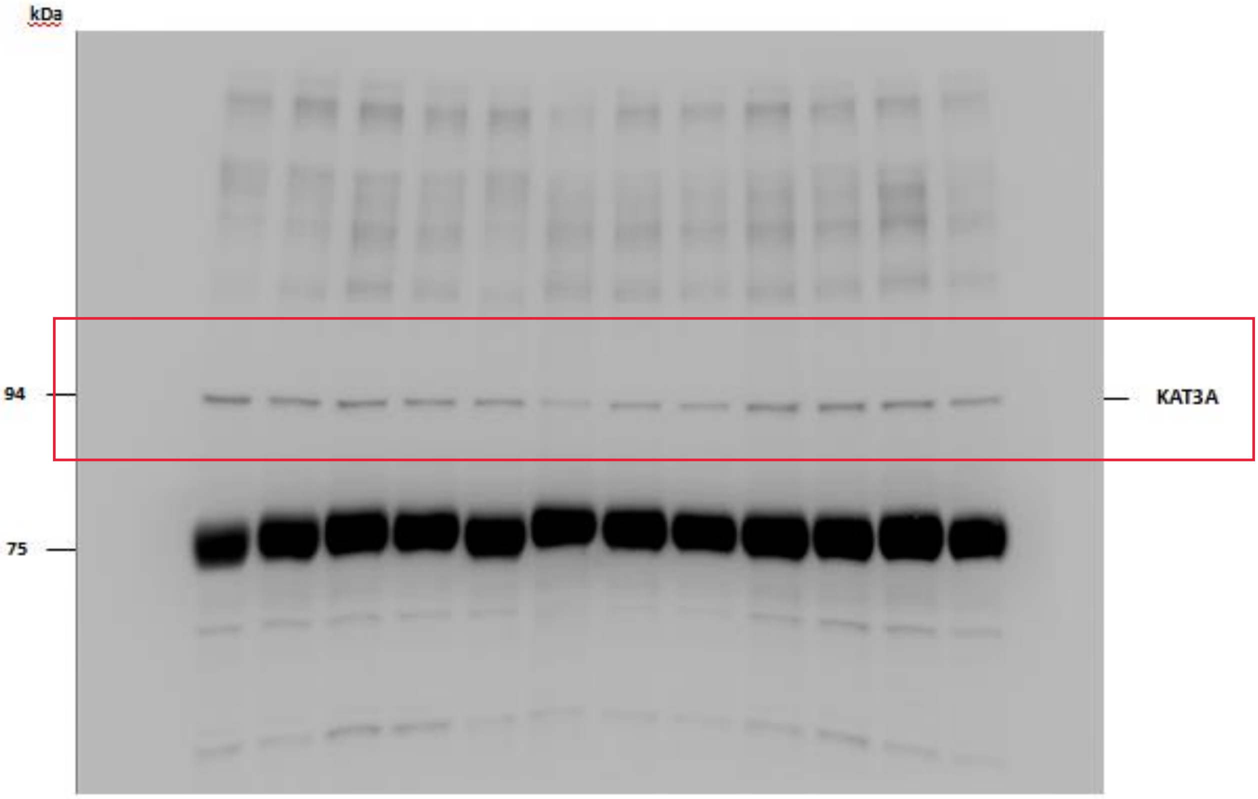

FIG. 2 KAT5

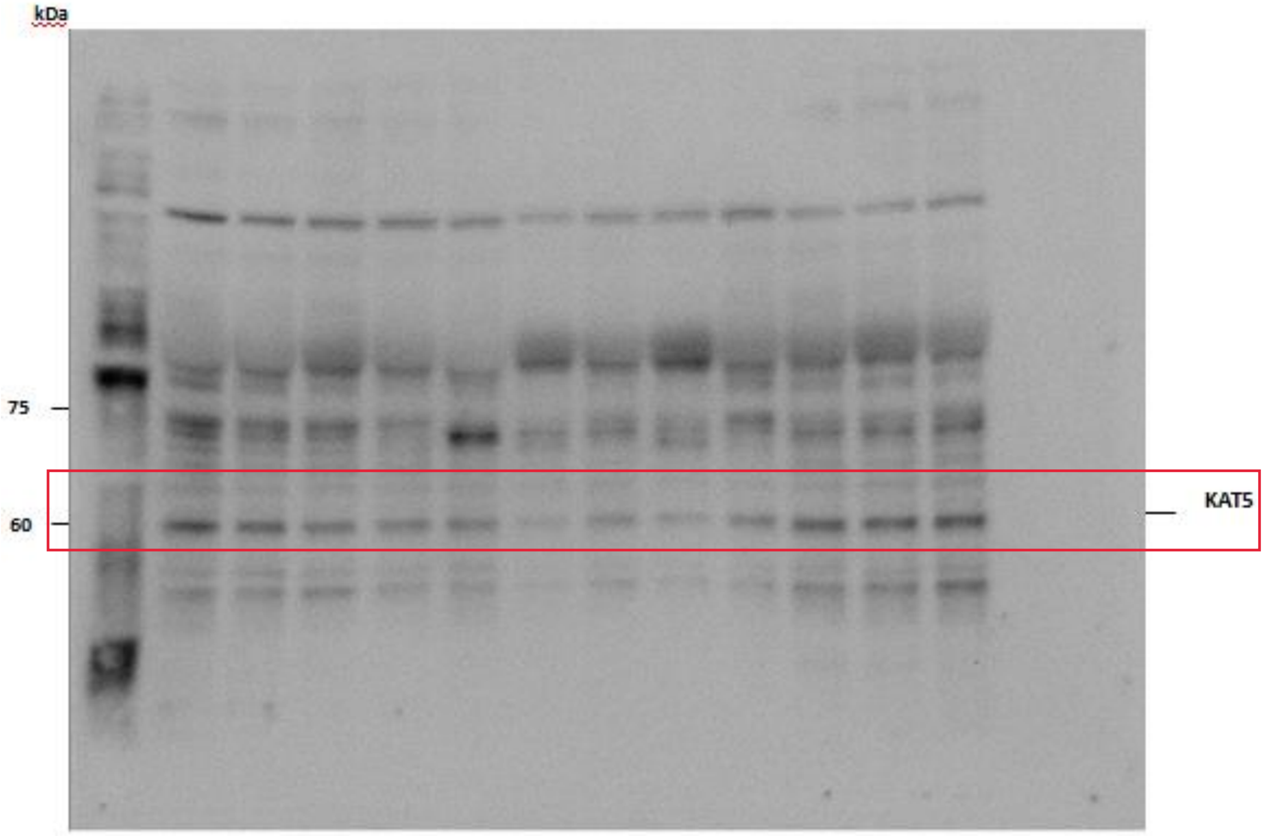

FIG. 2 KAT7

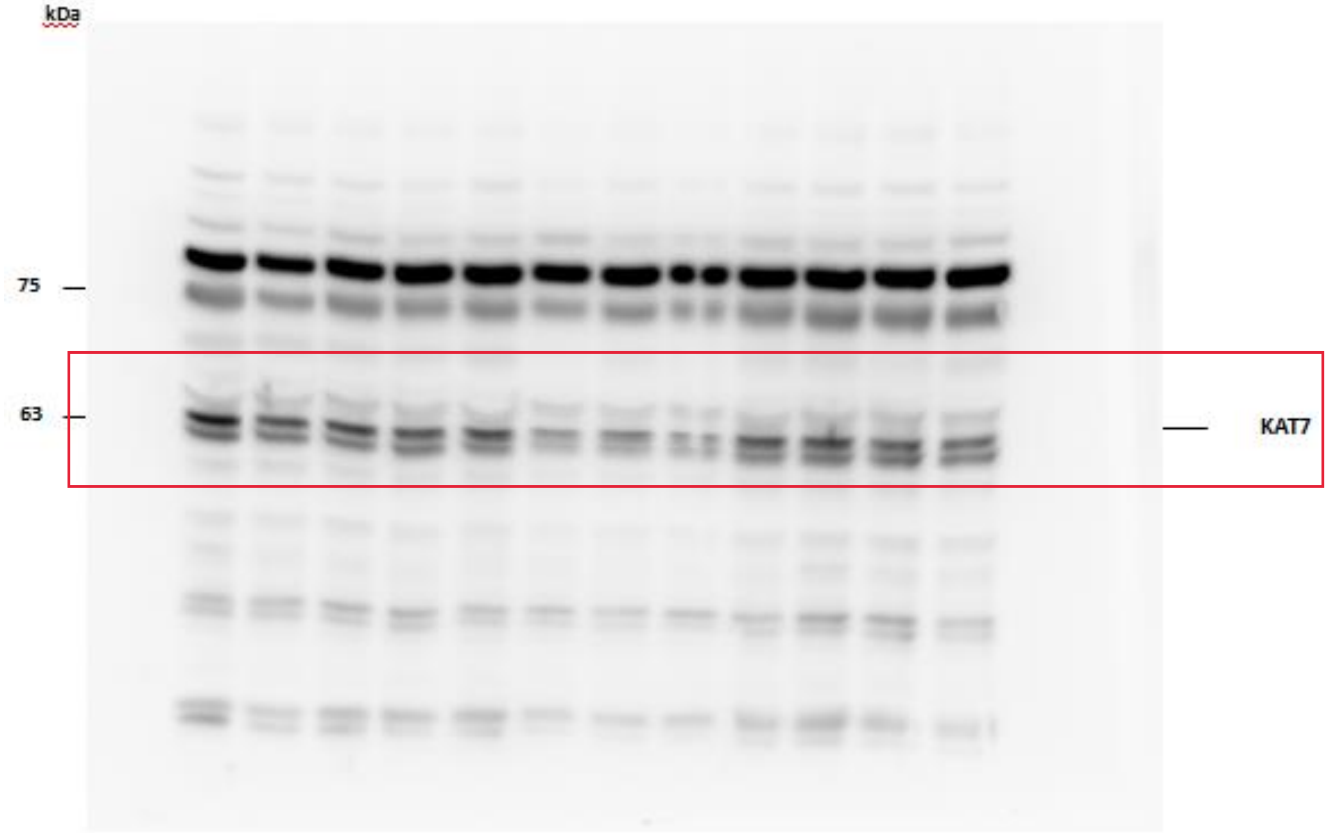

FIG. 2 KAT8

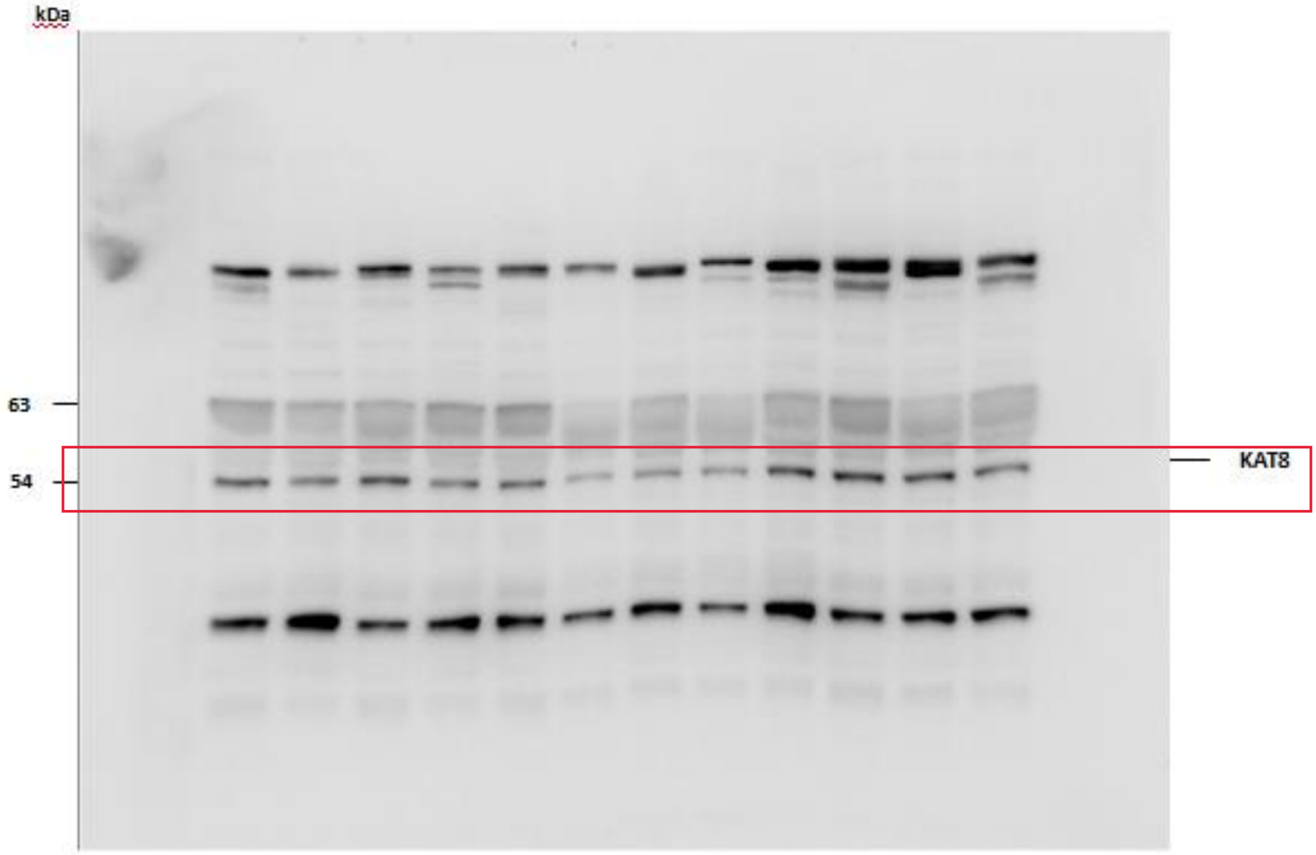

Figure 3 full blot images

FIG. 3 H2AK5ac

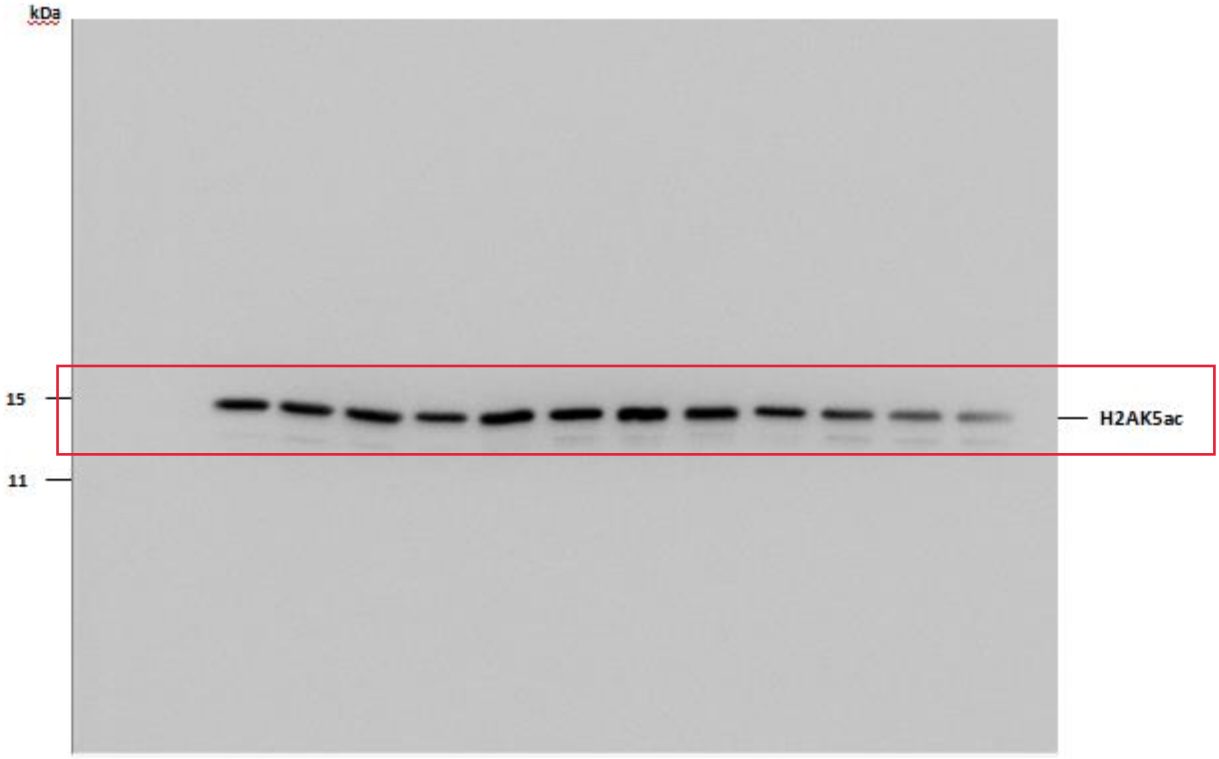

FIG. 3 H2BK5ac

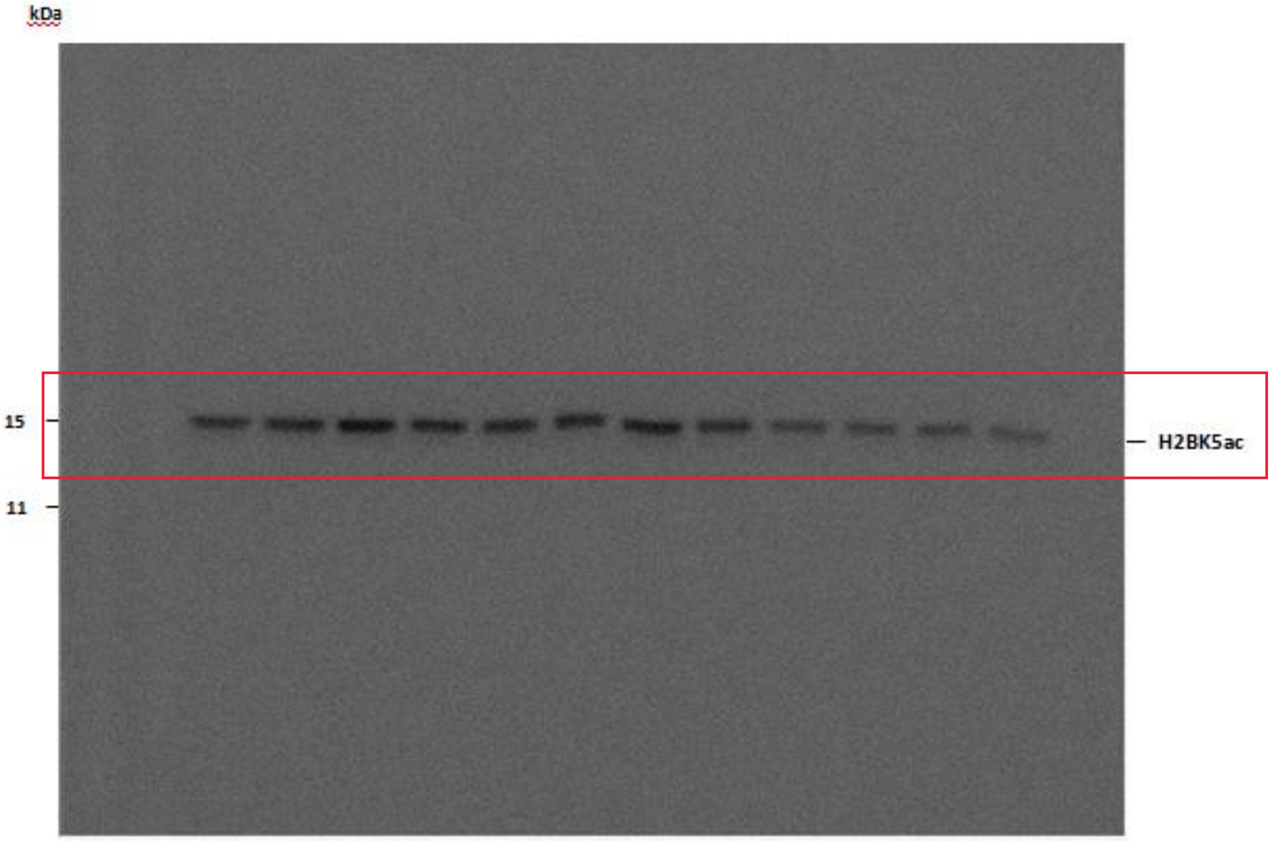

FIG. 3 H3K9ac

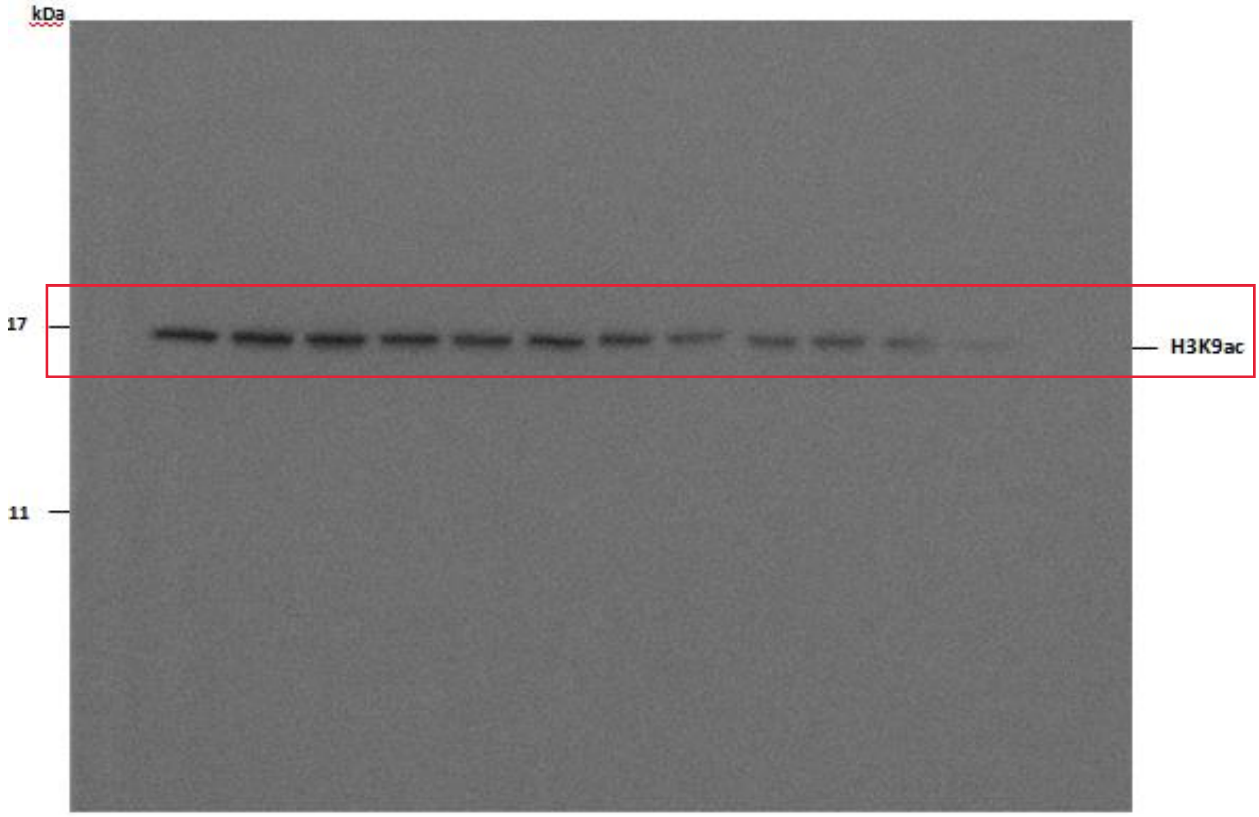

FIG. 3 H3K14ac

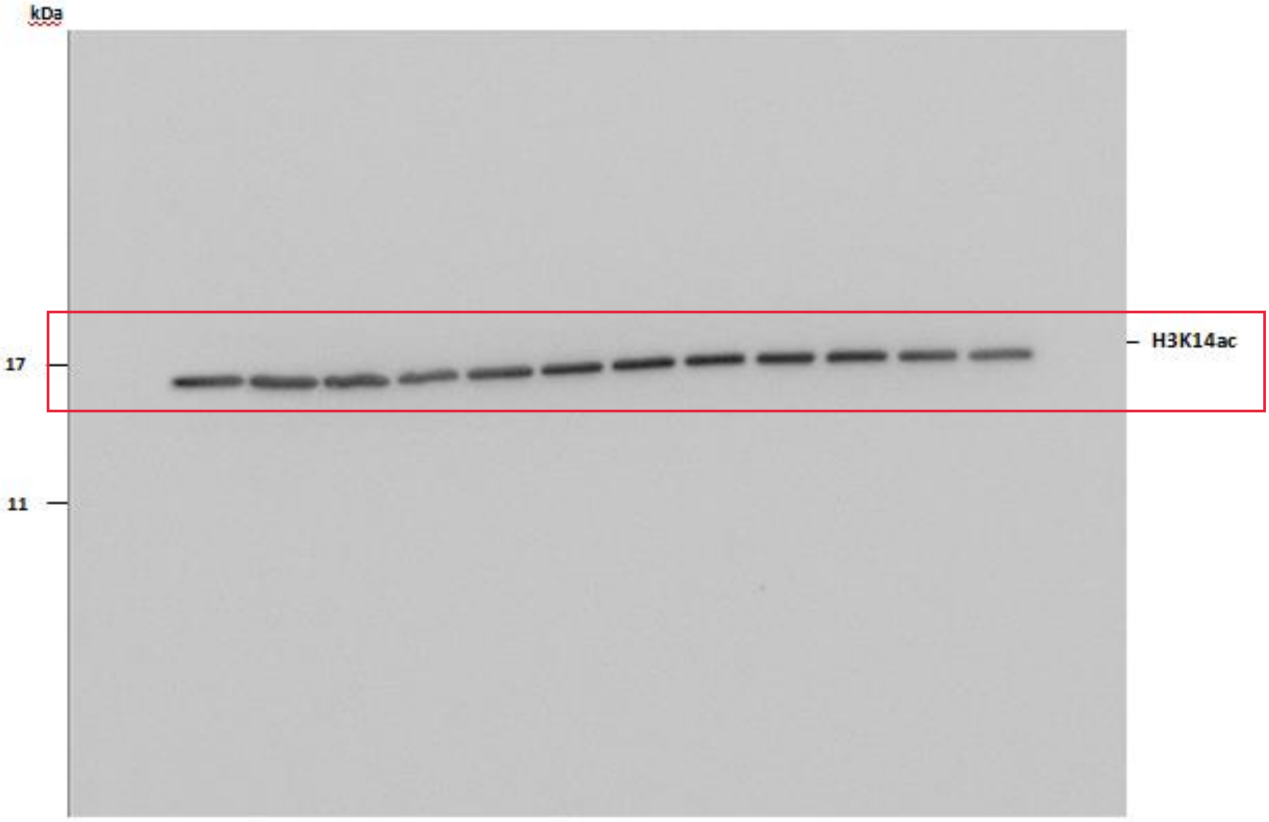

FIG. 3 H3K18ac

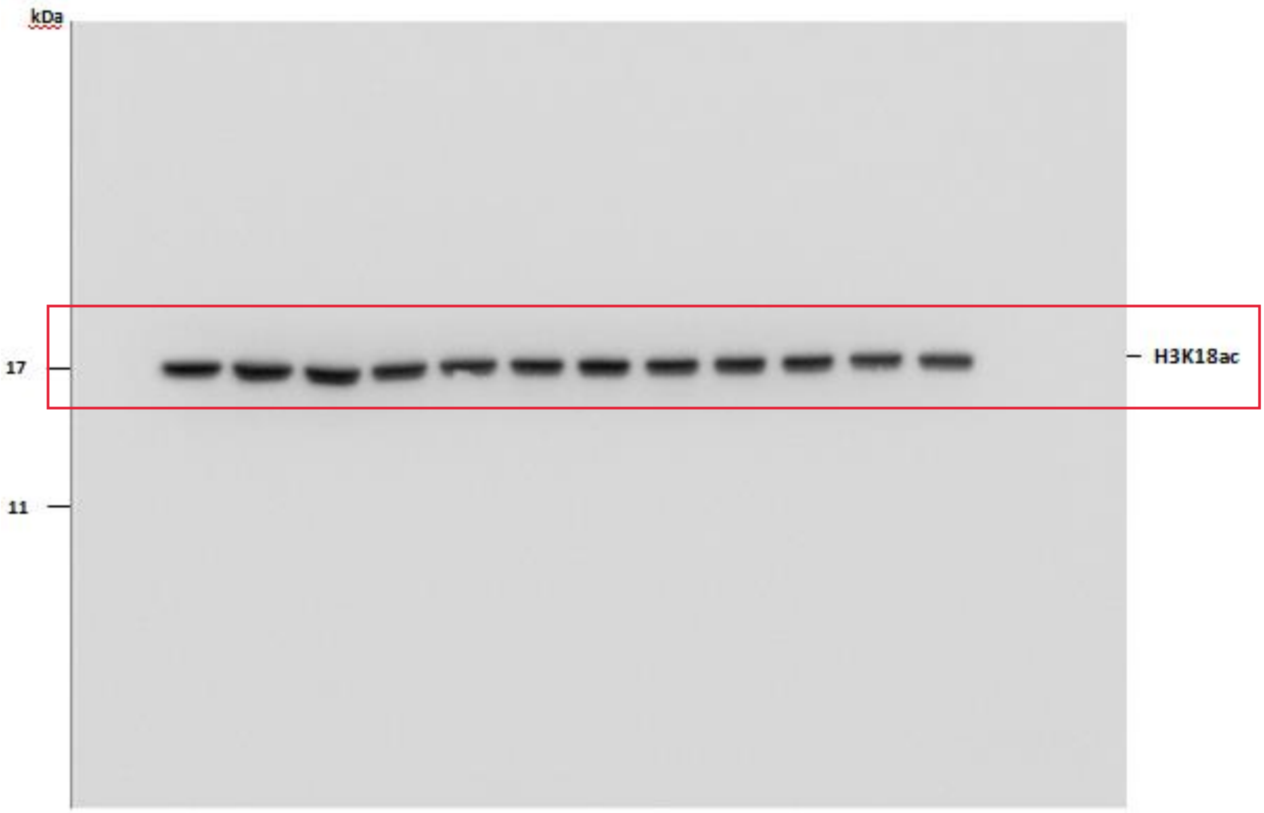

FIG. 3 H3K23ac

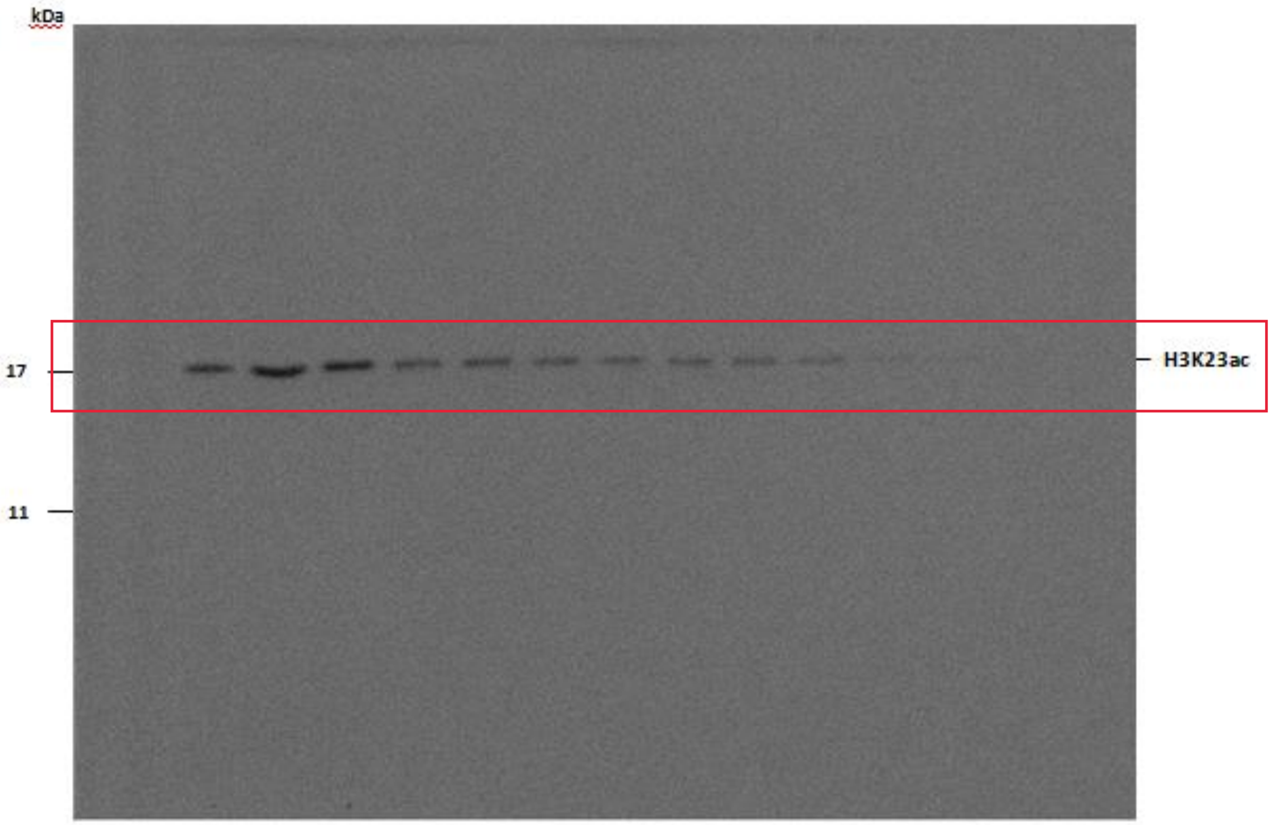

FIG. 3 H3K27ac

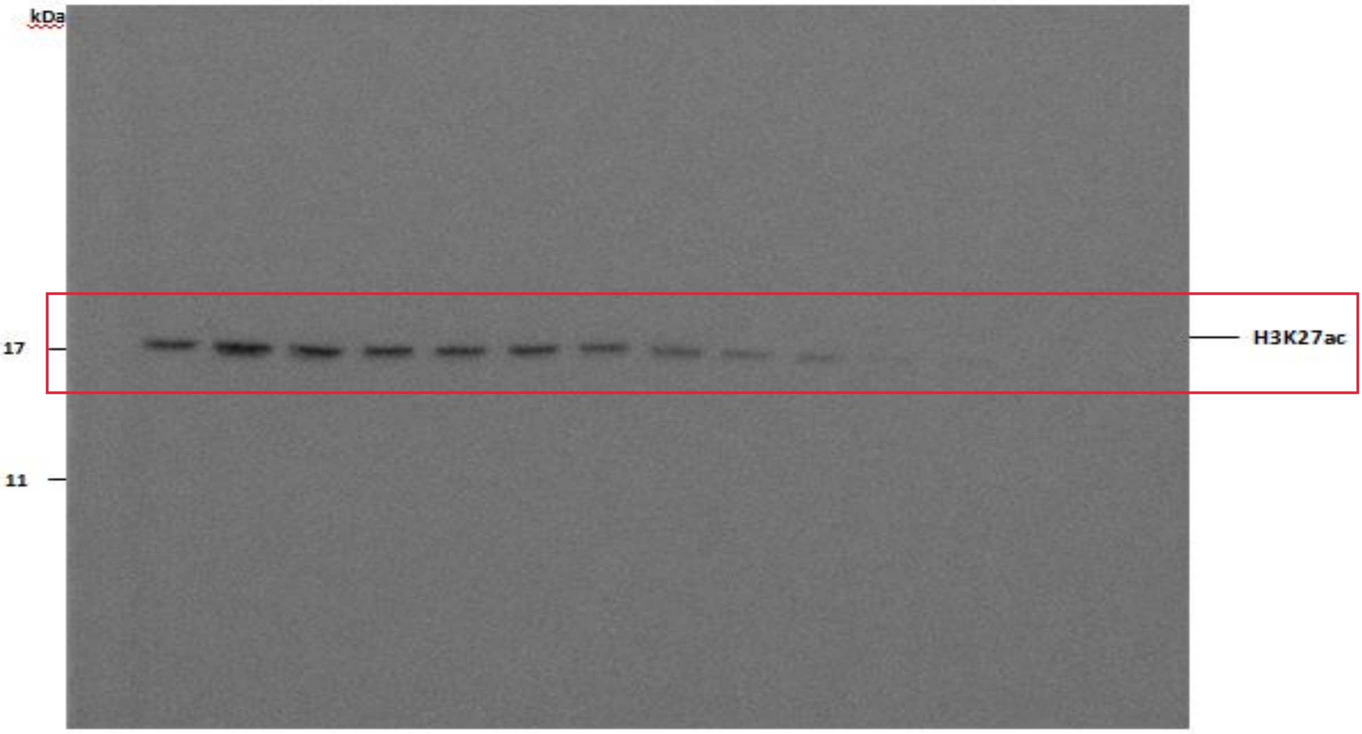

FIG. 3 H3K56ac

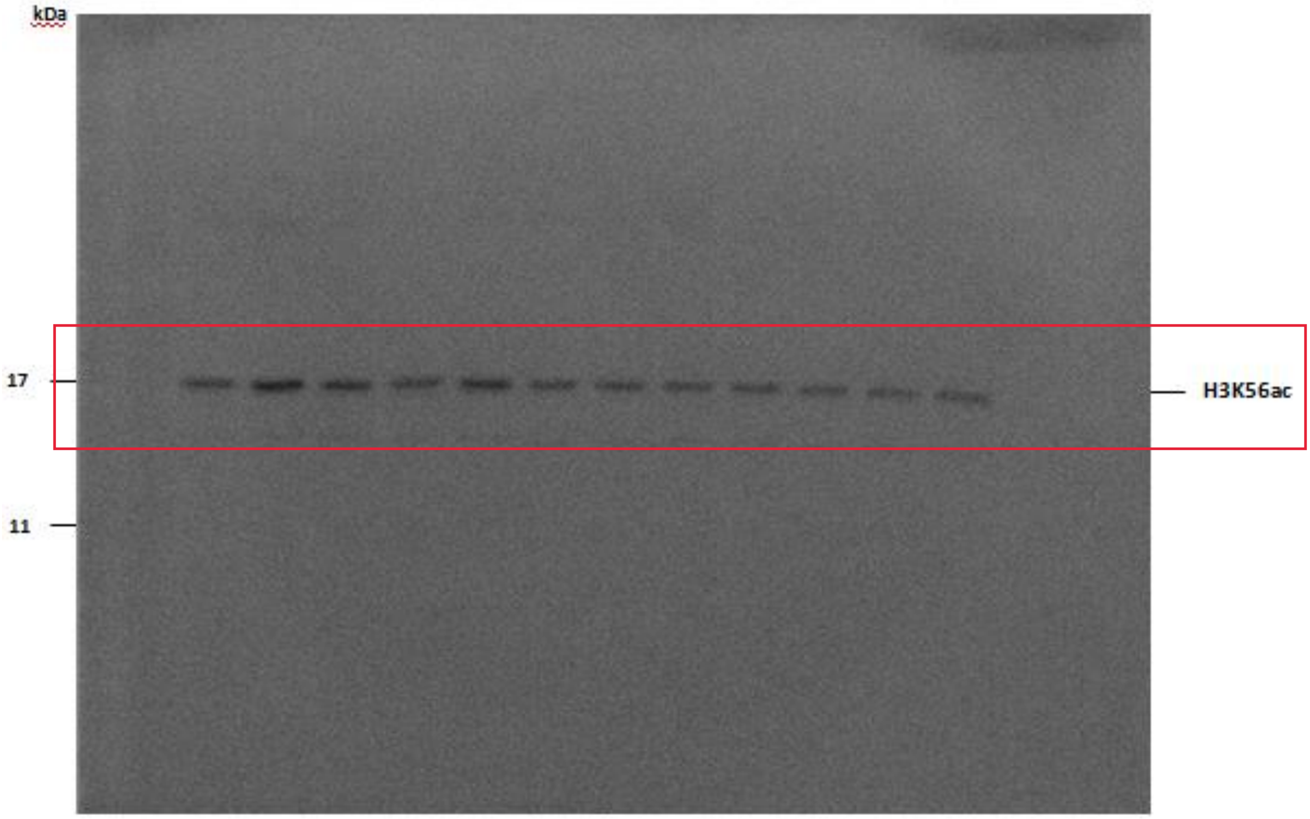

FIG. 3 H4K8ac

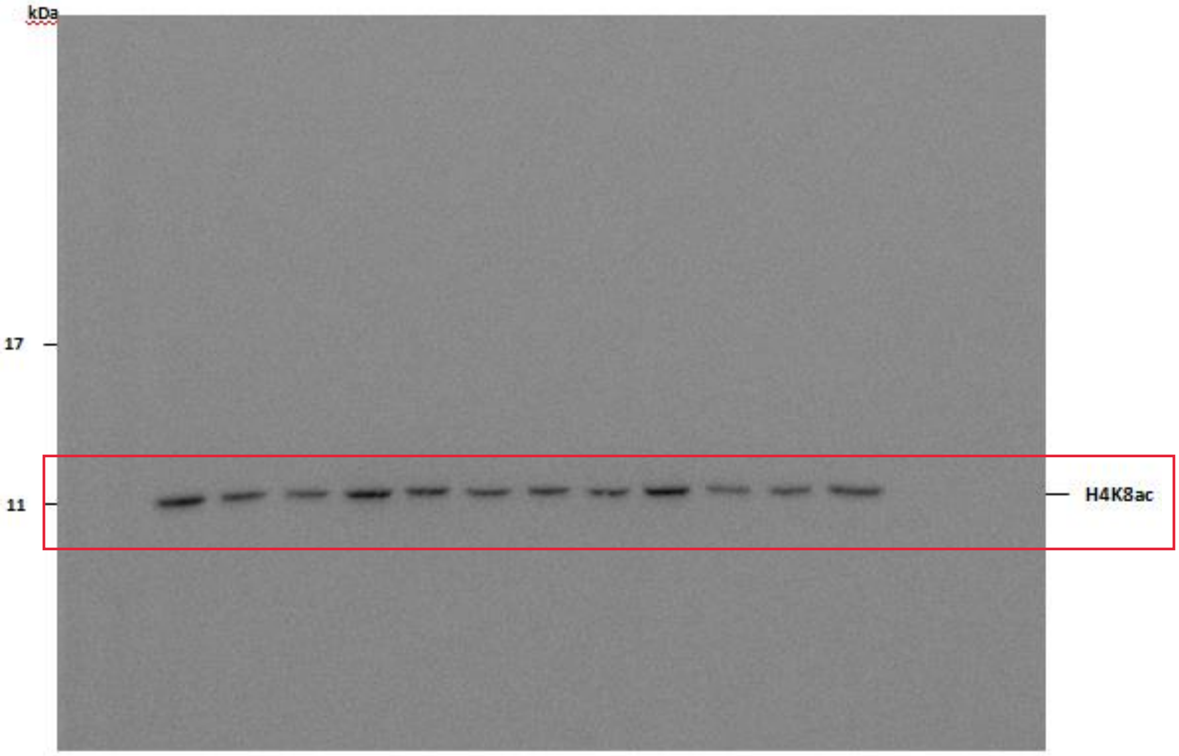

Figure 4 full blot images

FIG. 4 HDAC2

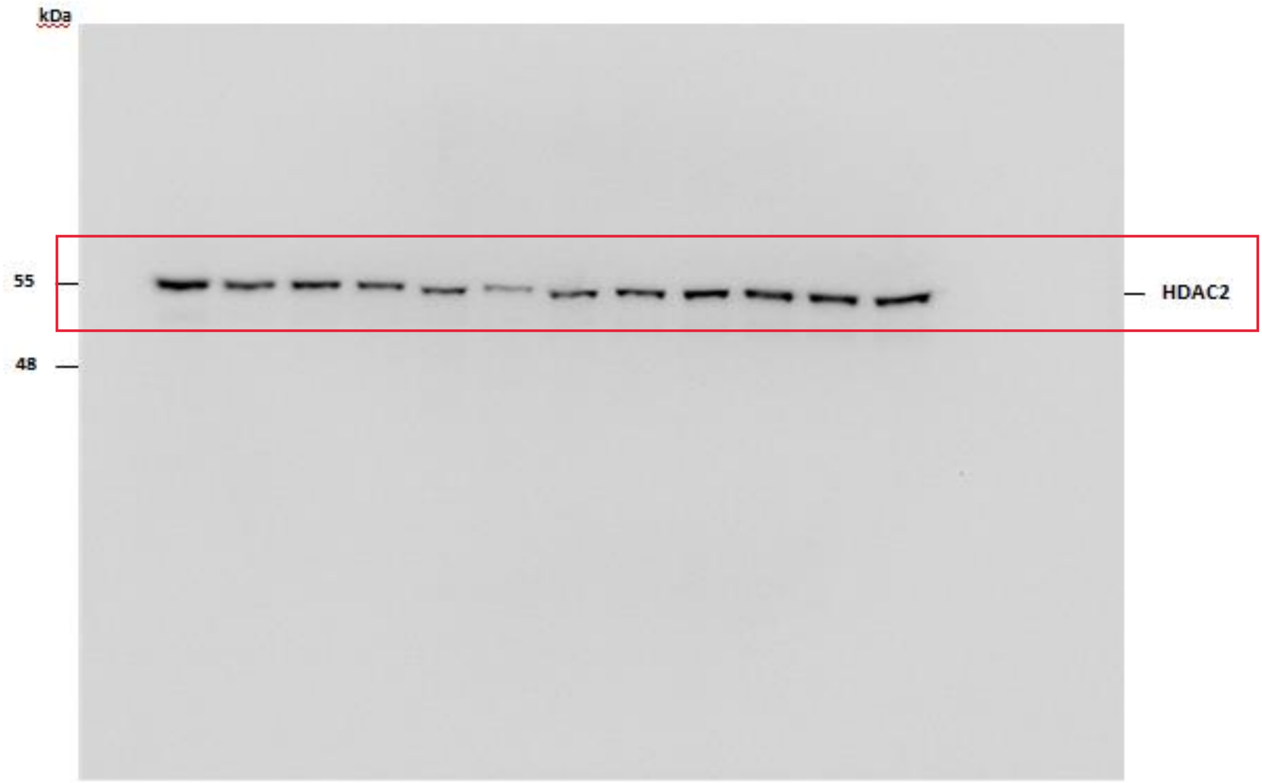

FIG. 4 HDAC3

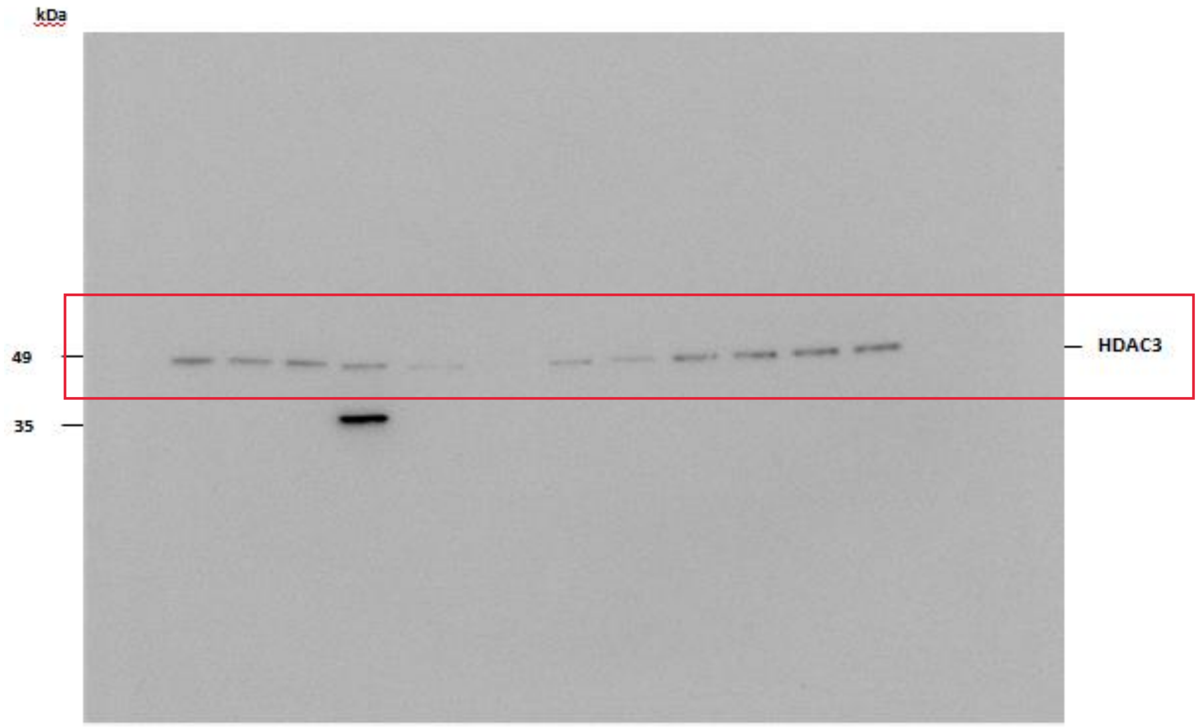

FIG. 4 HDAC4

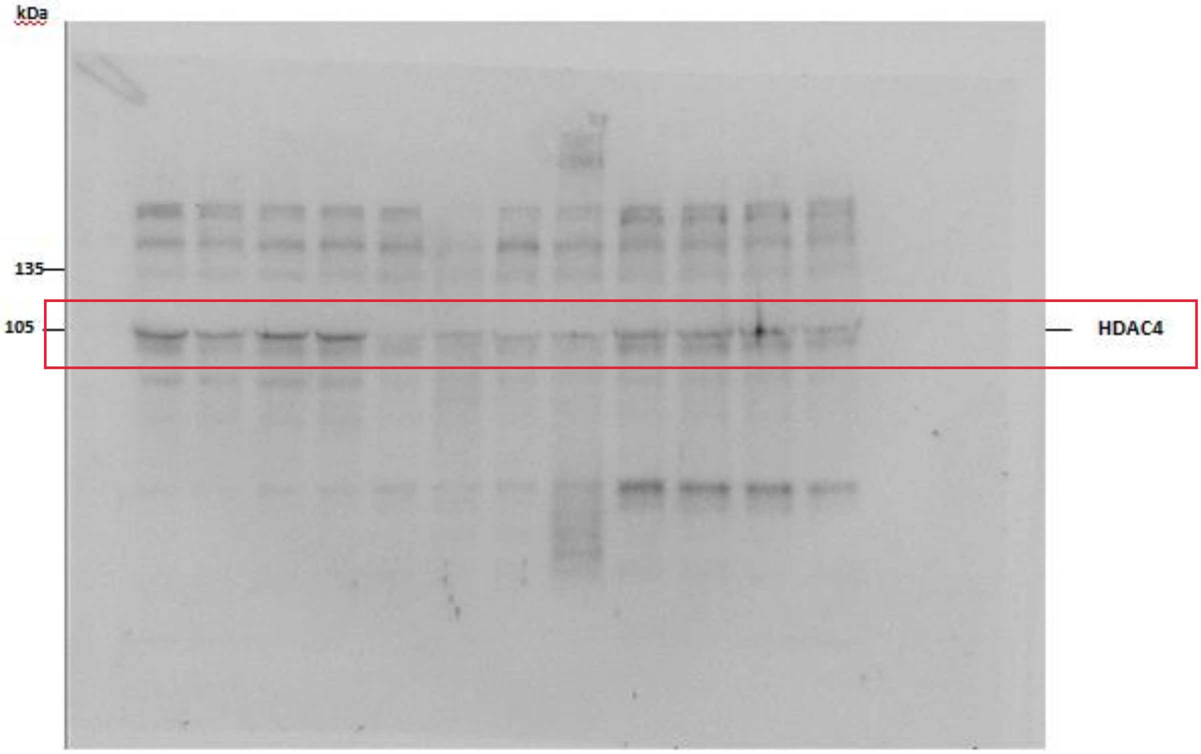

FIG. 4 HDAC5

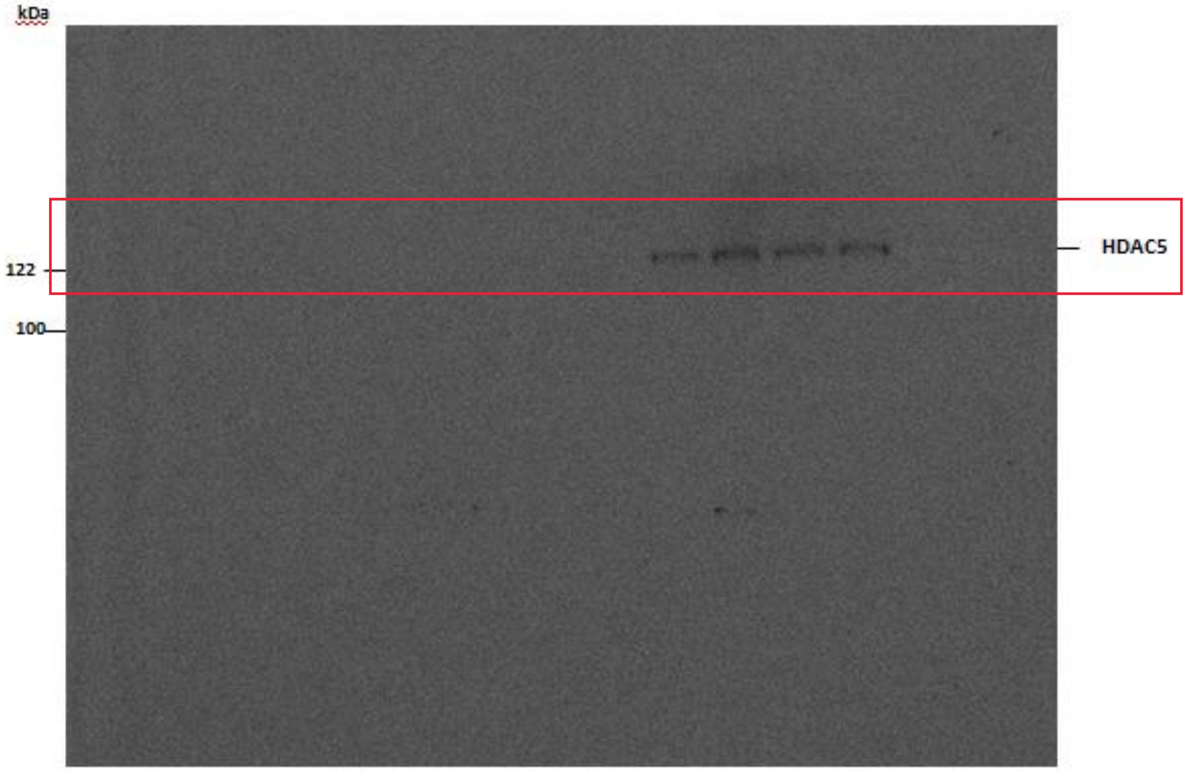

FIG. 4 HDAC6

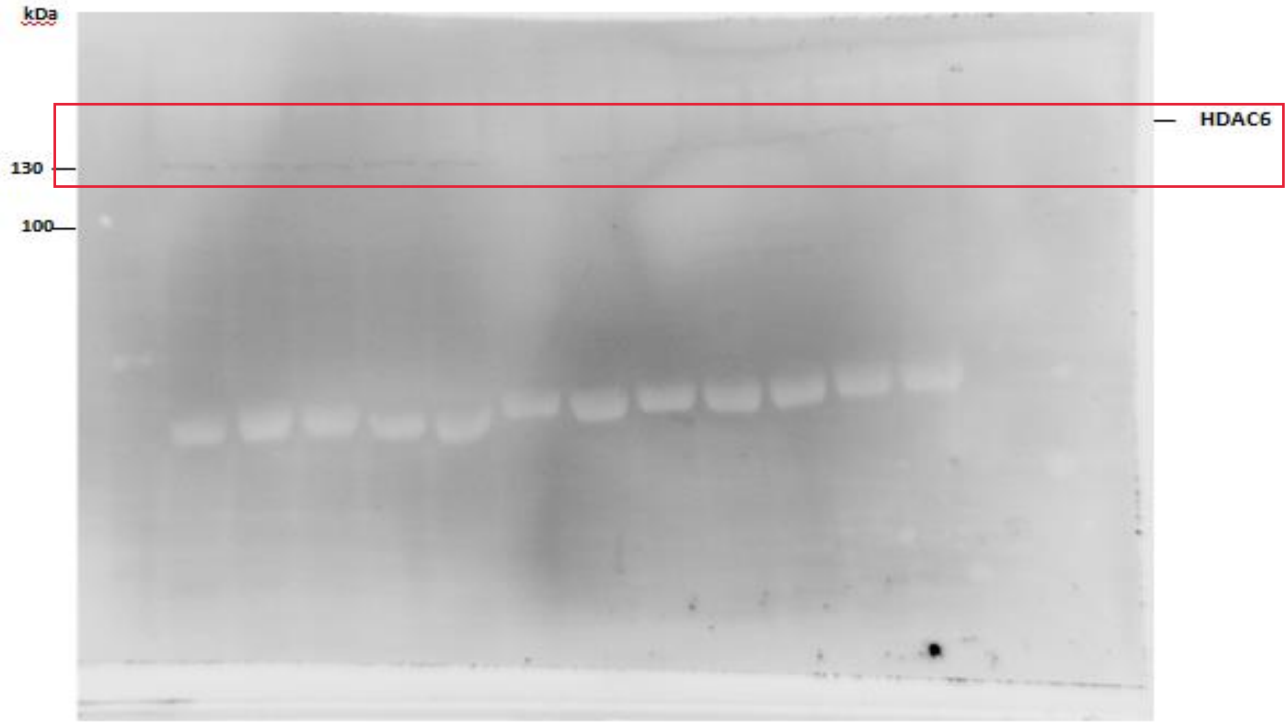

FIG. 4 HDAC8

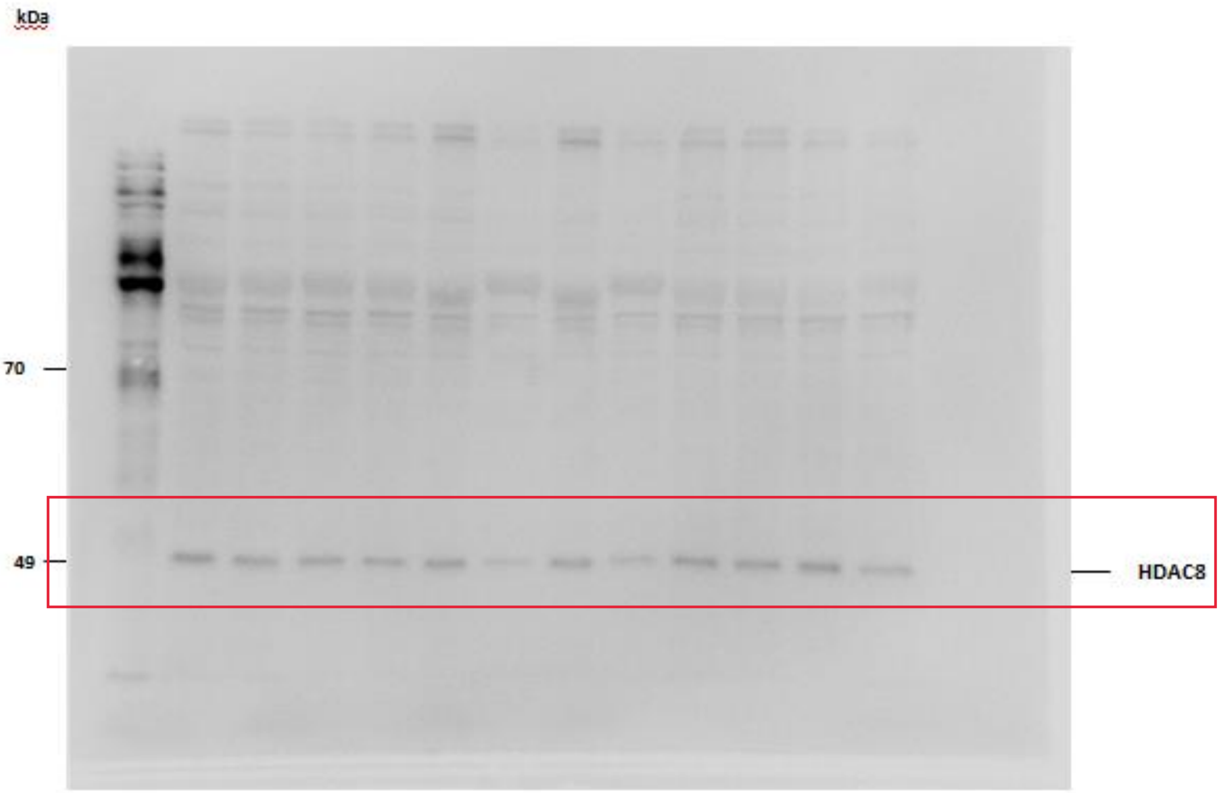

FIG. 4 HDAC10

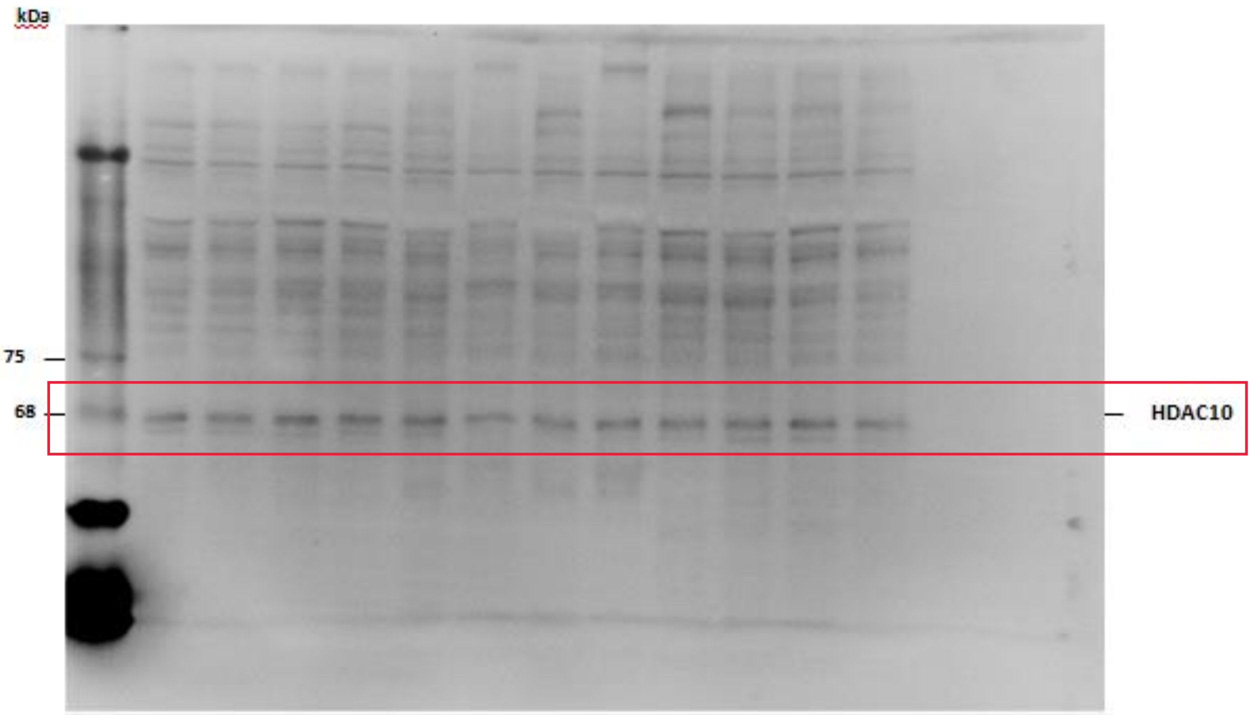

FIG. 4 HDAC11

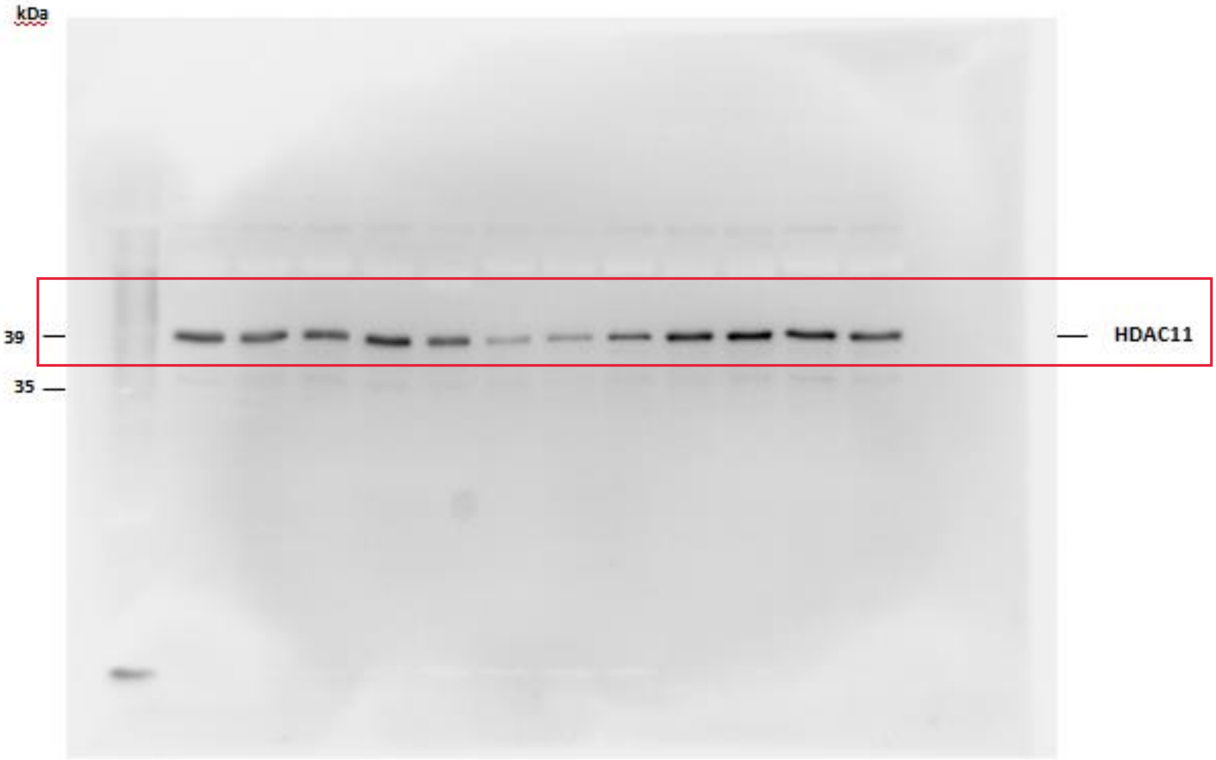

FIG. 4 p-HDAC4

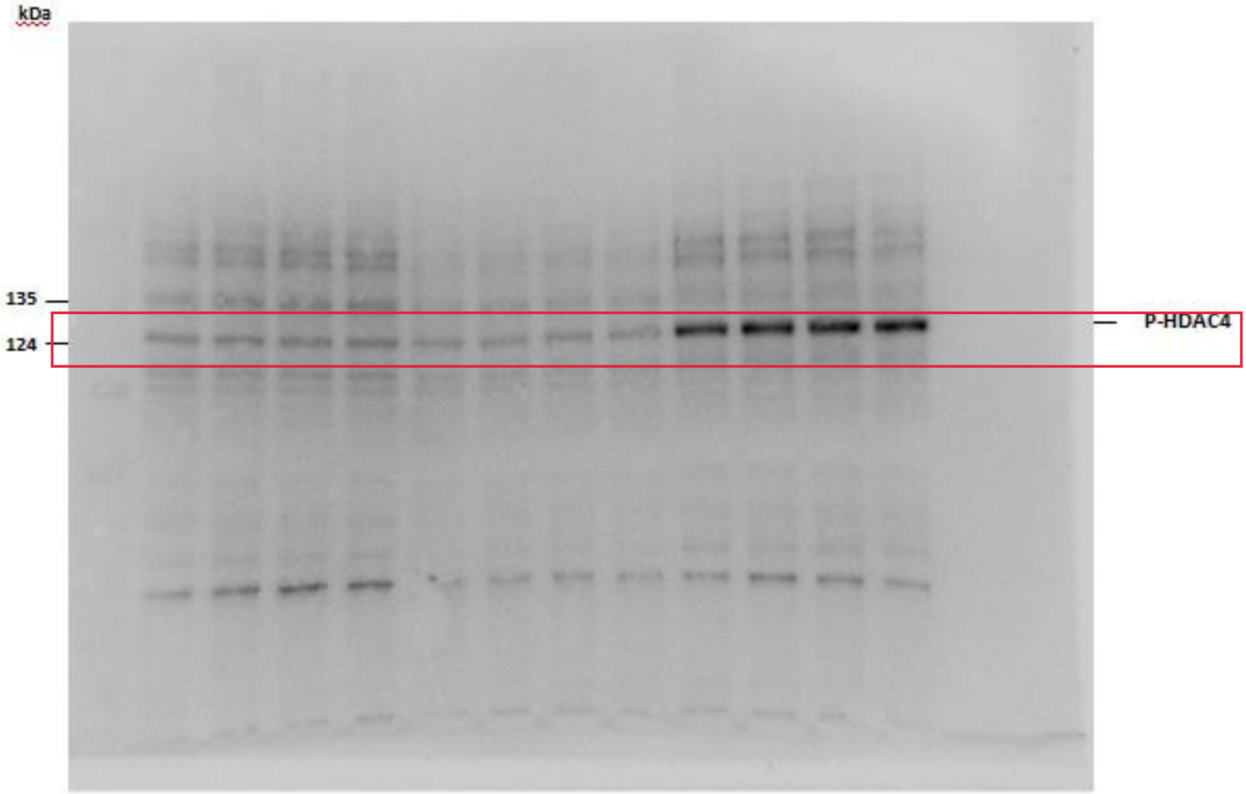

FIG. 4 p-HDAC8

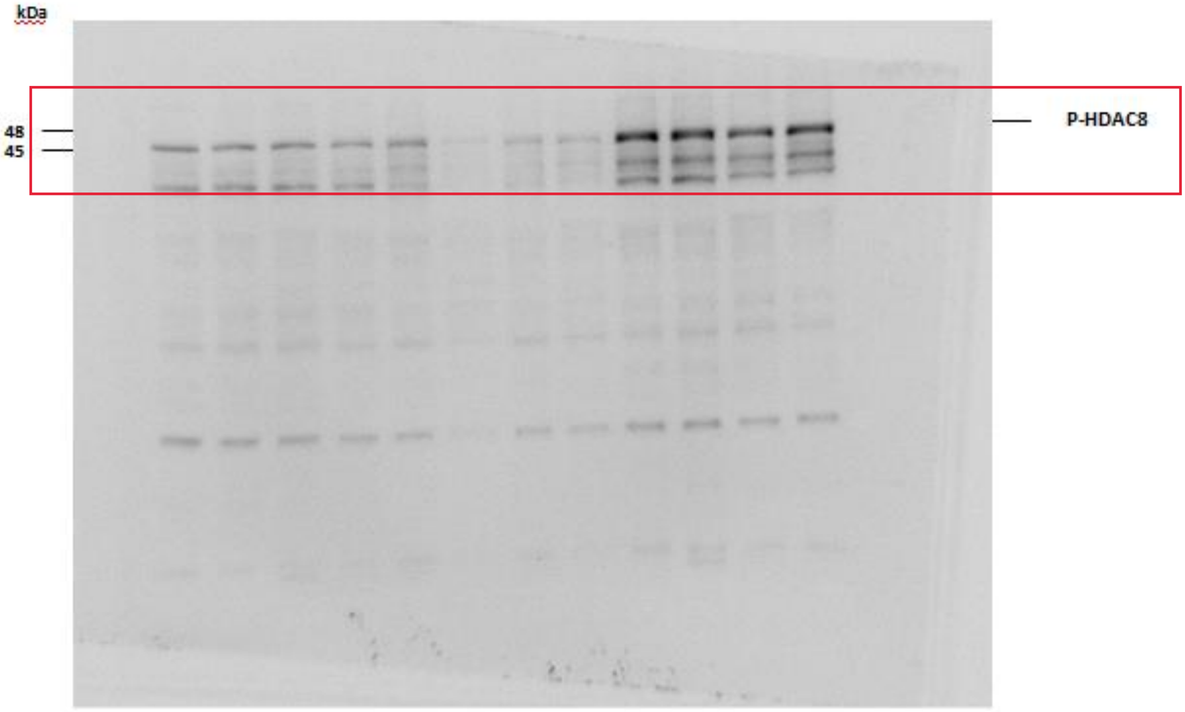

Supplement: Supplementary file 2 — Supplementary Material 2 [file 41598_2025_9521_MOESM2_ESM.pdf]
