# Supplementary material for: Contribution of reversible histone acetylation to freeze tolerance and recovery in wood frog kidneys
Source: Sci Rep. 2025 Jul 26;15:27243. doi: 10.1038/s41598-025-09521-x (PMC12297572; doi:10.1038/s41598-025-09521-x)
Supplement: Supplementary file 3 — Supplementary Material 3 [file 41598_2025_9521_MOESM3_ESM.pdf]

Supplemental Files S2

protein sequence alignment

```
frog_KAT2B              MSEAEGV-SAVVAQGSPAG----------GGGRGAESPAGGG-AAPIRIAAKKGQLRSAP    48
frog_KAT2A              MAELVQASQSRSSLQSPAGGQSATTTAAAGSSSGSSDPARPGLSQQQRASQRKAQVRALP    60
Human_KAT2A_C-term      ------------------------------------------------------------    0

frog_KAT2B              RAKKLEKLGVYSACKADETCKCNGWKNPNPPPTPPRMDLQQTVVSLTEPCRSCSHTLASH    108
frog_KAT2A              RAKKLEKLGVFSACKANDVCKCNGWKNPNPQ-TAPRMDLQQPAASLSEPCRSCGHSLADH    119
Human_KAT2A_C-term      ------------------------------------------------------------    0

frog_KAT2B              VSHLENVAEEEINRLLGIALDVEYLFTCVHKEEDADTKQVYFYLFKLLRKCILQMGKPVV    168
frog_KAT2A              VSHLENVSEDEINRLLGMVVDVENLFMSVHKEEDTDTKQVYFYLFKLLRKCILQMIRPVV    179
Human_KAT2A_C-term      ------------------------------------------------------------    0

frog_KAT2B              EGSLESPPFEKPSIEQGVNNFVQYKFSHLPAKERQTILELAKMFLNRINYWHLETPSQRR    228
frog_KAT2A              EGSLGSPPFEKPNIEQGVLNFVQYKFSHLQPKERQTMYELSKMFLLCLNYWKLETPSQFR    239
Human_KAT2A_C-term      ------------------------------------------------------------    0

frog_KAT2B              QRSQTEDIAAYKVNYTR-------------------------------------------    245
frog_KAT2A              QRSQNEDVANYKVNYTRWLCYCHVPQSCDSLPRYETTQVFGRSLLRSIFTVTRRQLLEKF    299
Human_KAT2A_C-term      ------------------------------------------------------------    0

frog_KAT2B              ---------------------FLSMLEEEVYSPNSPIWDEDFMSSSSRTSELGIQAVITN    284
frog_KAT2A              RVEKDKLVPEKRTLILTHFPKFLSMLEEEIYGETSPIWEADFTVPNTEVPQLVSRPAAVN    359
Human_KAT2A_C-term      ------------------------------------------------------------    0

frog_KAT2B              PPVSRTIPYVASPSSVDQSNSGSLSPSGKPA-LESSLGDKRKSSEPYSAEESKRPRTVGD    343
frog_KAT2A              TTAAPSAPLFS----NSLNNNSTLTSNGDAGISEPLPGEKRKLSDSLTIEDAKRIRVMGD    415
Human_KAT2A_C-term      ------------------------------------------------------------    0

frog_KAT2B              IPIELINEVMSTITDPAAMLGPETSFLSAISARDEAARLEERRGVIEFHVVGNSLNQKPN    403
frog_KAT2A              IPMELVNEVMLTITDPAAMLGPDTSLLSANAARDETARLEERRGIIEFHVIGNSLSQKSN    475
Human_KAT2A_C-term      ------------------------------------------------------------    0

frog_KAT2B              KKIMIWLVGLQNVFSHQLPRMPKEYITRLVFDPKHKTLALIKDGRVIGGICFRMFPSQGF    463
frog_KAT2A              KKILMWLVGLQNVFSHQLPRMPKEYITRLVFDPKHKTLALIKDGRVIGGICFRMFPTQGF    535
Human_KAT2A_C-term      ------------------------------------------------------------    0

frog_KAT2B              TEIVFCAVTSNEQVKGYGTHLMNHLKEYHIKHNVLNFLTYADEYAIGYFKKQGFSKDIKV    523
frog_KAT2A              TEIVFCAVTSNEQVKGYGTHLMNHLKEYHIKHNILYFLTYADEYAIGYFKKQGFSKDIKV    595
Human_KAT2A_C-term      ------------------------------------------------------------    0

frog_KAT2B              PKARYVGYIKDYEGATLMGCELNPRIPYTEFSVIIKKQKEIIKKMIERKQAQIRKVYPGL    583
frog_KAT2A              PKSRYLGYIKDYEGATLMECELNPRIPYTELSHIIKKQKEIIKKLIERRQAQIRKVYPGL    655
Human_KAT2A_C-term      ------------------------------------------------------------    0

frog_KAT2B              SCFKEGVRQIPIESIPGIRETGWKPSIKEKSKEPKDPEQLYNTLKNILQQVKSHQSAWPF    643
frog_KAT2A              TCFKEGVRQIPVECIPGIRETGWKPSCKEKGKEIKDPDQLYNMLKNLLAQIKSHPSAWPF    715
Human_KAT2A_C-term      ------------------------------------------------------------    0

frog_KAT2B              MEPVKRTDAPGYYEVIRFPMDLKTMSERVKNKYYVTKKLFMADLQRIFTNCREYNPPESE    703
frog_KAT2A              MEPVKKSEAPDYYEVIRFPIDLKTMTERLKNRYYVTKKIFIADLQRIITNCREYNPPDSE    775
Human_KAT2A_C-term      ----------------------------------VTRKLFVADLQRVIANCREYNPPDSE    26
                                                          **:*:*:*****:::********:**

frog_KAT2B              YFKCANILEKFFYTKIKEAGLIEK            727
frog_KAT2A              YCKCANTLEKFFYFKLKEGGLIDK            799
Human_KAT2A_C-term      YCRCASALEKFFYFKLKEGGLIDK            50
                        * :**. ****** *:**.***:*
```
